# Mechanistic investigation of a D to N mutation in DAHP synthase that dictates carbon flux into the shikimate pathway in yeast

**DOI:** 10.1038/s42004-023-00946-x

**Published:** 2023-07-15

**Authors:** Huayi Liu, Qingjie Xiao, Xinxin Wu, He Ma, Jian Li, Xufan Guo, Zhenyu Liu, Yan Zhang, Yunzi Luo

**Affiliations:** 1 Frontiers Science Center of Synthetic Biology and Key Laboratory of Systems Bioengineering (Ministry of Education), School of Chemical Engineering and Technology, Tianjin University, Tianjin, 300072 China; 2 Georgia Tech Shenzhen Institute, Tianjin University, Tangxing Road 133, Nanshan District, Shenzhen, 518071 China; 3 National Facility for Protein Science in Shanghai, Shanghai Advanced Research Institute (Zhangjiang Laboratory), Chinese Academy of Sciences, Shanghai, 201210 China; 4 Tianjin Key Laboratory for Modern Drug Delivery & High-Efficiency, Collaborative Innovation Center of Chemical Science and Engineering, School of Pharmaceutical Science and Technology, Tianjin University, Tianjin, 300072 China

**Keywords:** Metabolic pathways, Synthetic biology, Biosynthesis, X-ray crystallography, Enzymes

## Abstract

3-deoxy-D-*arabino*-heptulosonate-7-phosphate synthase (DAHPS) is a key enzyme in the shikimate pathway for the biosynthesis of aromatic compounds. _L_-Phe and _L_-Tyr bind to the two main DAHPS isoforms and inhibit their enzyme activities, respectively. Synthetic biologists aim to relieve such inhibitions in order to improve the productivity of aromatic compounds. In this work, we reported a point mutant of yeast DHAPS, Aro3^D154N^, which retains the wild type enzyme activity but converts it highly inert to the inhibition by _L_-Phe. The Aro3 crystal structure along with the molecular dynamics simulations analysis suggests that the D154N mutation distant from the inhibitor binding cavity may reduce the binding affinity of _L_-Phe. Growth assays demonstrated that substitution of the conserved D154 with asparagine suffices to relieve the inhibition of _L_-Phe on Aro3, _L_-Tyr on Aro4, and the inhibitions on their corresponding homologues from diverse yeasts. The importance of our discovery is highlighted by the observation of 29.1% and 43.6% increase of yield for the production of tyrosol and salidroside respectively upon substituting *ARO3* with *ARO3*^*D154N*^. We anticipate that this allele would be used broadly to increase the yield of various aromatic products in metabolically diverse microorganisms.

## Abstract (web summary)

3-Deoxy-D-arabino-heptulosonate-7-phosphate synthase (DAHPS) is a key enzyme in the shikimate pathway for the biosynthesis of aromatic compounds, however, its activity is inhibited by L-Phe and L-Tyr. Here, the authors report the D154N point mutation of yeast DAHPS, which retains the wild type enzyme activity but relieves the inhibition by L-Phe, thus increasing the production of downstream aromatic amino acid derivatives.

## Introduction

Plants and microbes use the shikimate pathway as the biosynthetic route to produce aromatic amino acids (AAAs), while animals rely on acquisition of essential AAAs from their foods. Therefore, the prevalence of the shikimate pathway rivals that of nitrogen fixation and photosynthesis. More importantly, the shikimate pathway is employed by plants and microbes as early steps where they branch out to synthesize a great variety of natural products with aromatic ring components, with many of them being highly valuable and widely used in foods, nutraceuticals, cosmetics, and pharmaceuticals such as phenylethanoids, resveratrol, flavonoids and alkaloids^1–4^.

**Fig. 1:**
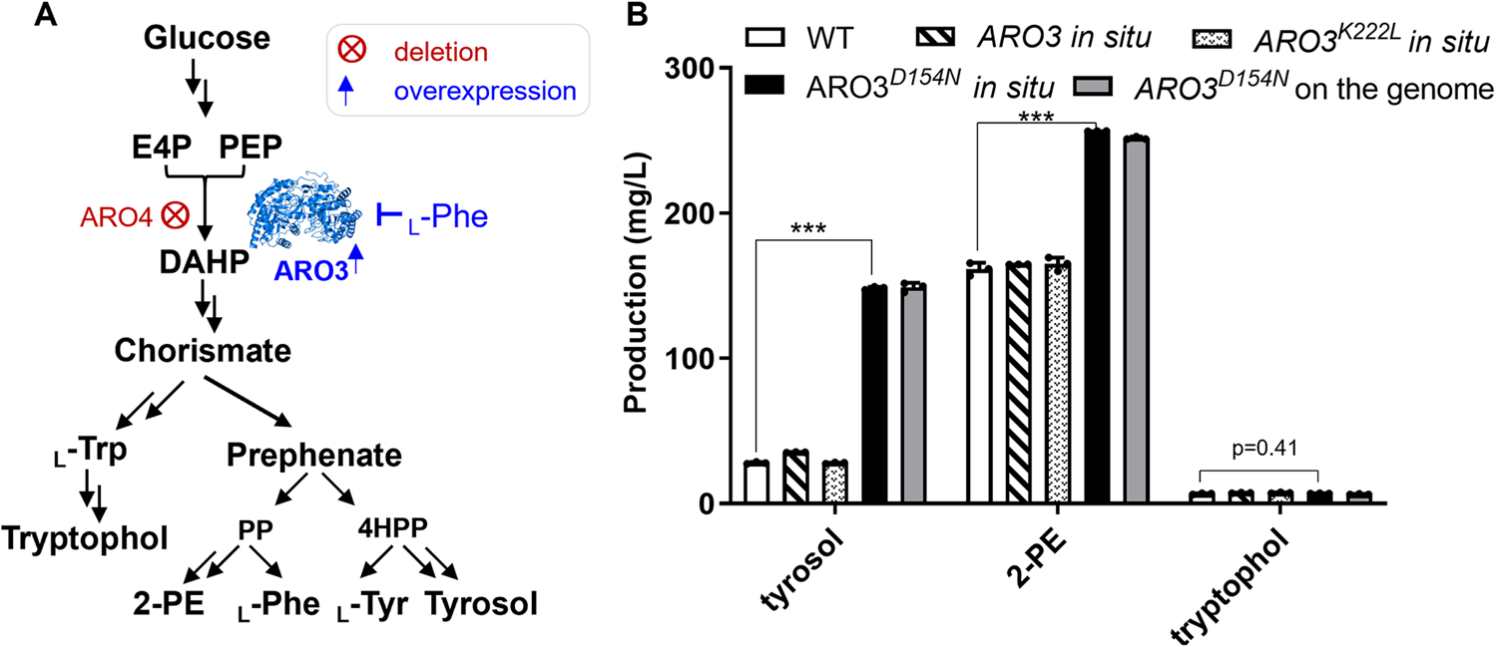
The impact of the overexpression of *ARO3* mutants on the production of aromatic amino acid derivatives. **A** The biosynthetic pathways of tryptophol, tyrosol, and 2-phenylethanol. The double arrows represent multiple enzymatic steps. Metabolite abbreviations: PEP, phosphoenol pyruvate; E4P, erythrose 4-phosphate; DAHP, 3-deoxy-D-*arabino*-heptulosonate-7-phosphate; _L_-Trp, _L_-tryptophan; _L_-Tyr, _L_-tyrosine; _L_-Phe, _L_-phenylalanine; 4-HPP: 4-hydroxyphenylpyruvate; PP: phenylpyruvate; 2-PE, 2-phenylethanol. **B** The extracellular concentrations of tryptophol, tyrosol, and 2-PE through the overexpression of *ARO3*, *ARO3*^*K222L*^, and *ARO3*^*D154N*^ in situ or through the addition copy of *ARO3*^*D154N*^ on the genome. Error bars represent the standard deviation (±SD) of three biological repeats. Source data are available in Supplementary Data 4. ***p < 0.001.

In the seven-step shikimate pathway, the first committed step involves an aldol condensation of PEP and E4P to form DAHP, catalyzed by DAHPS, which can be classified into Type I and Type II enzymes based on their sequence relationships^5^. Type I DHAPS is further divided into Type Iα and Iβ. Commonly used model organisms and industrial fermentation strains such as *Escherichia coli* (*E. coli*) *and Saccharomyces cerevisiae* (*S. cerevisiae*) contain Type Iα DHAPS, which is allosterically regulated and feedback inhibited by downstream AAAs^1^. For example, the two isomeric Type Iα DAHPSs, Aro3/Aro4 of *S. cerevisiae* and AroG/AroF of *E. coli*, are feedback inhibited by _L_-phenylalanine (_L_-Phe) and _L_-tyrosine (_L_-Tyr), respectively (Fig. 1A)^6–8^.

Overexpression of Aro4^K229L^, a mutant resistant to the feedback inhibition of _L_-Tyr, has been commonly used as a strategy to successfully unlock the carbon flux into the shikimate pathway, resulting in higher yields of aromatic products in engineered *S. cerevisiae* strains^1,7,9^. However, the beneficial effects of the analogous Aro3 mutant, *ARO3*^*K222L*^ have been controversial. Significantly increased production of protocatechuic acid has been observed^10^, while no notable increase of the yields of tyrosol, salidroside, 2-phenylethanol, and chlorogenic acid have been reported by others and us^11–13^. The detailed mechanism that how Aro4^K229L^ could relieve the feedback inhibition of _L_-Tyr remains unclear. Nevertheless, the discrepancy indicates that Aro3 and Aro4 differ in the responses to the inhibitors of their own and that Aro3^K222L^ may not fully relieve the feedback inhibition of _L_-Phe in *S. cerevisiae*.

In search for an Aro3 mutant that is insensitive to the feedback inhibition of _L_-Phe to overcome the bottleneck for the flux into the shikimate pathway, we noticed that a point mutation D146N located apart from the inhibitor binding pocket of AroG has been commonly used to relieve the feedback inhibition of _L_-Phe to overproduce aromatic products in *E. coli*^1,8,14^. Inspired by this, Aro3^D154N^ that is an equivalent to the D146N mutant of the *E. coli* isozyme AroG was designed. Structural, computational, and biochemical characterization of this mutant unravels its mechanism in efficiently eliminating the feedback inhibition of _L_-Phe on Aro3 in yeast. This mechanism also explains our observation that an analogous mutation to the conserved Asp in Aro4 or other homologs from various yeast strains works equally well in alleviating the feedback inhibition either from _L_-Tyr or _L_-Phe, including non-conventional industrial yeasts. The generality expands the enzyme toolbox for production of diverse aromatic compounds in a great variety of microbial cell factories, including model hosts and non-conventional hosts. To demonstrate its potential, we introduced this allele to engineered *S. cerevisiae* strains that overproduce tyrosol and salidroside respectively, resulting in further significantly increased yields.

## Results

### Relieve of the feedback inhibition on Aro3 by overexpression of Aro3^D154N^ in *S. cerevisiae*

Aro3^K222L^ and Aro3^D154N^ were constructed and used to substitute the wild-type Aro3 of *S. cerevisiae* in situ, respectively. The promoter of *ARO3* was exchanged to the constitutive *TEF1* promoter to remove the transcription regulation^15^. To assess Aro3 mutants without interference by Aro4, Aro3 mutants were overexpressed in *aro4Δ* background strain A3_1 (Supplementary Table 1). The extracellular titers of aromatic fusel alcohols, tyrosol, 2-phenylethanol, and tryptophol, belonging to the _L_-Tyr, _L_-Phe, and _L_-Trp branch products respectively, were measured as indicatives of the carbon flux of AAAs biosynthesis pathway^7^ (Fig. 1). Aro3^K222L^ overexpressing did not increase the yield of any of the three compounds (Fig. 1B). By contrast, Aro3^D154N^ significantly increased the titers of aromatic products (over 300% increase for tyrosol and 50% for 2-PE, no increase of tryptophol observed because of the *trp1Δ* mutation in the WT strains) (Fig. 1B). Furthermore, the titers of AAA derivatives in the A3_1 strain with a D154N mutation in the endogenous *ARO3* were comparable to those in the strain carrying the wild-type *ARO3* and an additional copy of *ARO3*^*D154N*^ integrated at a different locus of the genome, suggesting that this is a dominant mutant in abolishing the feedback inhibition (Fig. 1B).

**Fig. 2:**
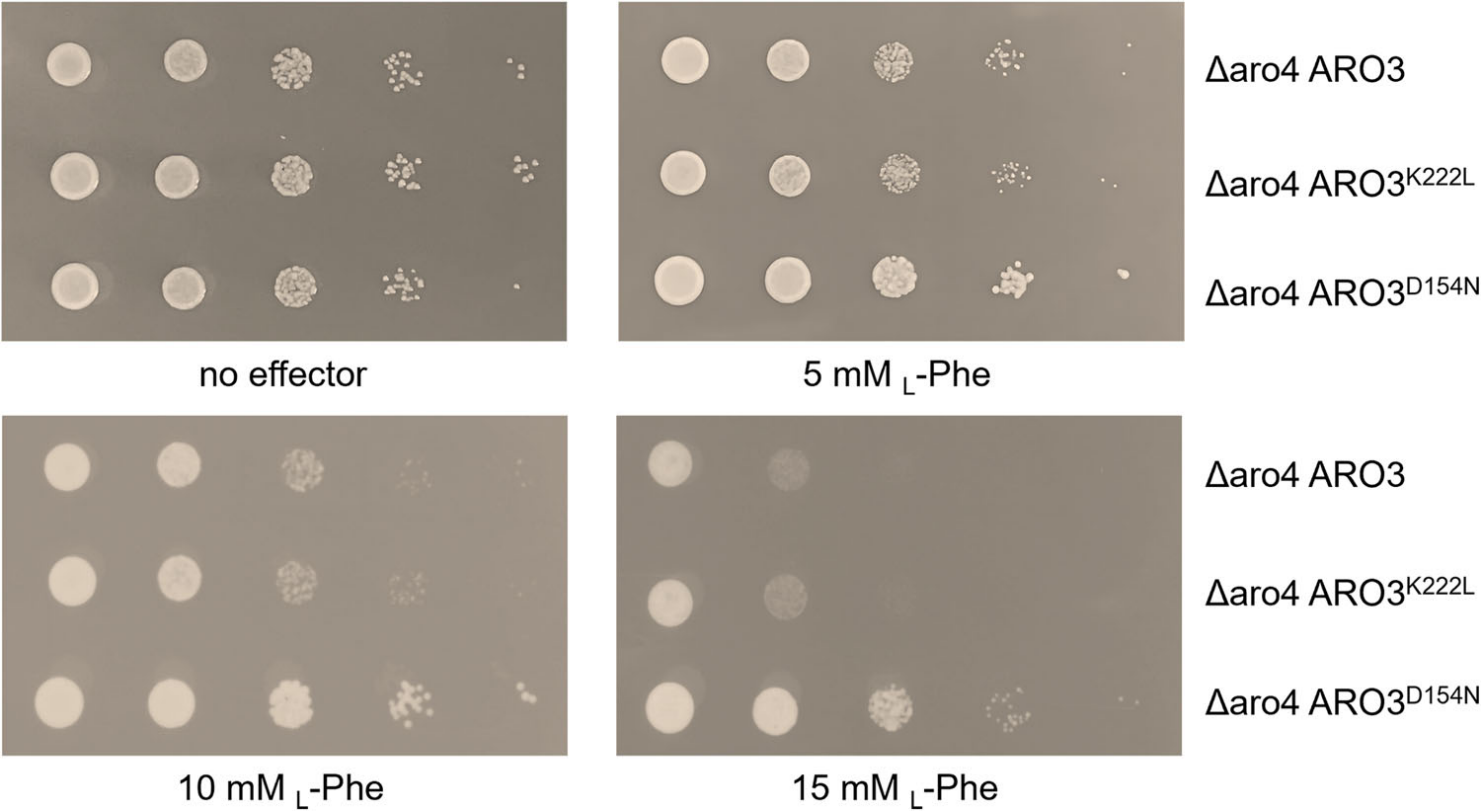
Growth of different yeast strains carrying various Aro3 mutants. To compare the effects of different Aro3 mutants, the yeast strains were cultivated in MV liquid medium, harvested at 36 h, diluted at OD_600_ = 1.0, and serially 10-fold diluted to spot onto the MV solid medium in the presence or absence of 5 mM, 10 mM, and 15 mM _L_-Phe. Plates were incubated at 30 °C for 60 h.

Next, we decided to test if the increased yield of aromatic fusel alcohols is a result of *ARO3*^*D154N*^ relieved from the feedback inhibition by _L_-Phe. Plate assays were performed with added aromatic amino acids to the growth media. The *TRP1* gene was repaired in A3_1 strain to resume _L_-Trp synthesis, generating A3_4 strain (CEN.PK2-1C *Δaro4 TRP1* P_TEF1_-*ARO3*). Growth of the parental A3_4 strain was inhibited by _L_-Phe, but not _L_-Tyr nor _L_-Trp (Fig. 2, Supplementary Figure 1). Such unique _L_-Phe sensitivity persists through the overexpression of the *ARO3*^*K222L*^ allele but could be relieved by introducing the *ARO3*^*D154N*^ allele (Fig. 2 and Supplementary Figure 1).

### Enzyme activity of Aro3^D154N^

**Fig. 3:**
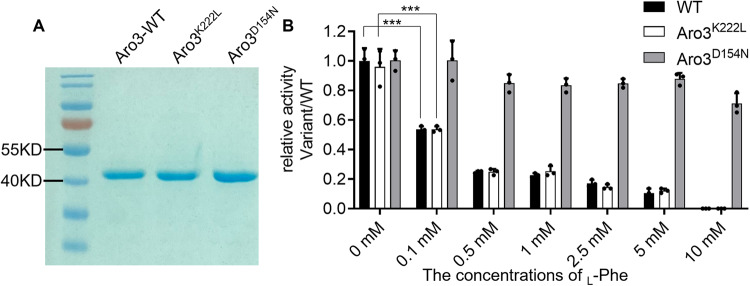
Activities of purified Aro3 enzymes in the presence and absence of L-phenylalanine. **A** The SDS-PAGE evaluation of recombinant proteins of Aro3, Aro3^K222L^, and Aro3^D154N^. See Supplementary Figure 2 for the uncropped SDS-PAGE image. **B** Relative activities of recombinant Aro3 variants in the presence or absence of _L_-Phe. The activities were measured in the presence of 0 to 10 mM of the _L_-Phe. Activity of the wild-type Aro3 without effector was set to 1.0. Error bars represent the standard deviation of three biological repeats. Source data are available in Supplementary Data 4. ***p < 0.001.

For in vitro biochemical and biophysical characterization of the enzymes, we recombinantly produced and purified Aro3 and its two variants, Aro3^K222L^ and Aro3^D154N^ (Fig. 3). The three enzymes exhibited comparable activity in the absence of _L_-Phe (Fig. 3B). Low concentration of _L_-Phe (0.1 mM) inhibited the enzyme activity of wild-type Aro3 and Aro3^K222L^ by half, but did not affect Aro3^D154N^ activity (Fig. 3B). High concentration of _L_-Phe (10 mM) completely abolished the activity of wild-type Aro3 and Aro3^K222L^, while Aro3^D154N^ retained the majority of its enzyme activity (71.2%) (Fig. 3B). Taken together, Aro3 activity is allosterically inhibited by _L_-Phe and we demonstrated that the inhibition could be rescued by a D154N single point mutation.

### Molecular basis of relieved allosteric inhibition

**Fig. 4:**
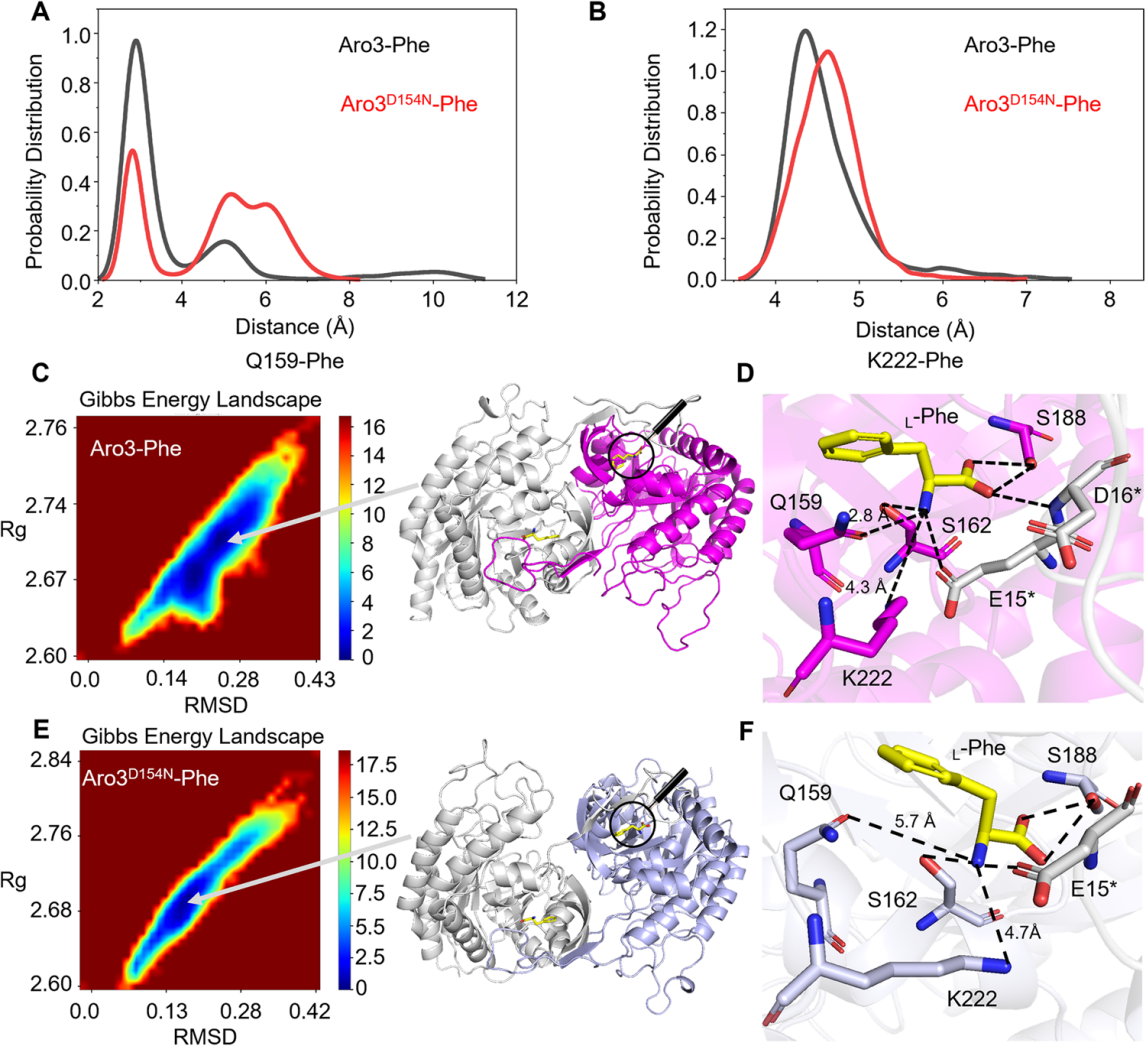
The molecular dynamics (MD) simulations analysis of Aro3-Phe and Aro3^D154N^-Phe. **A** Distribution of the distances between Q159 and _L_-Phe. **B** Distribution of the distances between K222 and _L_-Phe. Frequency distribution of the distances for the wild-type Aro3 (black) and Aro3^D154N^ (red). The distances are calculated using the carboxyl oxygen (OE1) of Q159, the nitrogen of the ε-amino group (NE) of K222 and the nitrogen (N) of _L_-Phe. The last 150 ns of simulation of each MD runs was used for analysis. **C**, **E** The free energy landscape (FEL) diagram as a function of RMSD of Cα atoms and Rg for the dimer of Aro3 as the two reaction coordinates of Aro3-Phe (**C**) and Aro3^D154N^-Phe (E). The typical snapshots from corresponding minimum energy wells were extracted. **D**, **F** Zoomed-in view of the distances between Q159/K222 and _L_-Phe of Aro3-Phe (**D**) and Aro3^D154N^-Phe (**F**) in the corresponding extracted conformation. The side chains of the _L_-Phe binding residues are shown as sticks and the residues marked as asterisk (*) belong to different subunits of Aro3 dimer. The _L_-Phe was shown as yellow sticks. Oxygen and nitrogen are shown in red and blue respectively. Black dotted lines indicate hydrogen bonds.

To further investigate the mechanism of the D154N mutation in relieving the inhibition by _L_-Phe, we determined the crystal structure of Aro3 at 3.3 Å (Table 1). The Aro3 crystals contain one dimer per asymmetric unit with each monomer exhibiting a canonical (β/α)_8_ TIM-barrel fold common to other Type Iα DAHPS enzymes (Supplementary Figure 3)^6,14,16–20^. Next, we used the AutoDockTools-1.5.6 program to model the complex structures. Two available isozyme-ligand complex structures, AroG from *E. coli* (PDB code: 1KFL) and *Nm*DAHPS from *Neisseria meningitidis* (PDB code: 4UC5) are used for comparison with our docking model. The positioning of the Phe and its interacting residues of the two complex crystal structures and our docking model can be overlayed nicely (Supplementary Figure 4). Then the docking model was applied to performe unconstrained molecular dynamics (MD) simulations (1000 ns). After the RMSD of the C_α_ atoms reached equilibrium, the last 650 ns of each trajectory was subjected to further analysis (Supplementary Figure 5). During the simulations, _L_-Phe was harbored in a cavity formed by helices α3 and α4, β-sheets of β6a/β6b and their adjacent loops, and the N-terminus of the second subunit of the dimer. Strikingly, the distance between the carboxyl oxygen (OE1) of Q159 and the nitrogen (N) of _L_-Phe was centered around 2.8 Å in the wild-type complex, while a much longer distance (centered around 5.3 Å and 5.8 Å) was found in the Aro3^D154N^ mutant complex (Fig. 4A). The distance between the nitrogen of the ε-amino group (NZ) of K222 and the nitrogen of _L_-Phe was also increased from 4.3 Å in the wild-type complex to 4.7 Å in the Aro3^D154N^ mutant complex (Fig. 4B). Taken together, the hydrogen bonding between _L_-Phe with the two key residues (Q159 and K222) of Aro3 were weakened by introducing the D154N mutation. We computed the free energy landscape (FEL) of the wild-type and Aro3^D154N^ mutant complexes based on the radius of gyration (Rg) for the dimer of Aro3 and the RMSD of Cα atoms. In the extracted typical conformations of local energy minima based on FEL analysis, longer distances between the two key residues (K222 and Q159) of Aro3^D154N^ and the nitrogen of _L_-Phe were found in the mutant Aro3 complex (Fig. 4E, F, 4.7 Å/5.7 Å) compared to the corresponding distances in the wild-type complex (Fig. 4C, D, 4.2 Å/2.8 Å). Structural analyses suggested that the binding affinity for _L_-Phe was reduced by the single D154N point mutation.

**Table 1:** Data collection and refinement statistics (molecular replacement).

	Aro3
*Data collection*	
Space group	P 2 21 21
Cell dimensions	
* a*, *b*, *c* (Å)	73.68, 95.561, 105.785
* α*, *β*, *γ* (°)	90, 90, 90
Resolution (Å)	105.79–3.3 (3.56–3.3)^a^
*R*_sym_ or *R*_merge_	0.115 (1.503)
*I* / σ*I*	9.6 (1.3)
Completeness (%)	96.6 (95.9)
Redundancy	7.0 (7.3)
*Refinement*	
Resolution (Å)	34.37–3.3 (3.418–3.3)
No. reflections	11,322 (1145)
*R*_work_/*R*_free_	0.252/0.295
No. atoms	
Protein	5760
Ligand/ion	0
Water	0
*B*-factors	176.3
Protein	176.3
Ligand/ion	0
Water	0
R.m.s. deviations	
Bond lengths (Å)	0.013
Bond angles (°)	1.76

^a^Number of xtals for each structure should be noted in footnote. Values in parentheses are for highest-resolution shell.

**Fig. 5:**
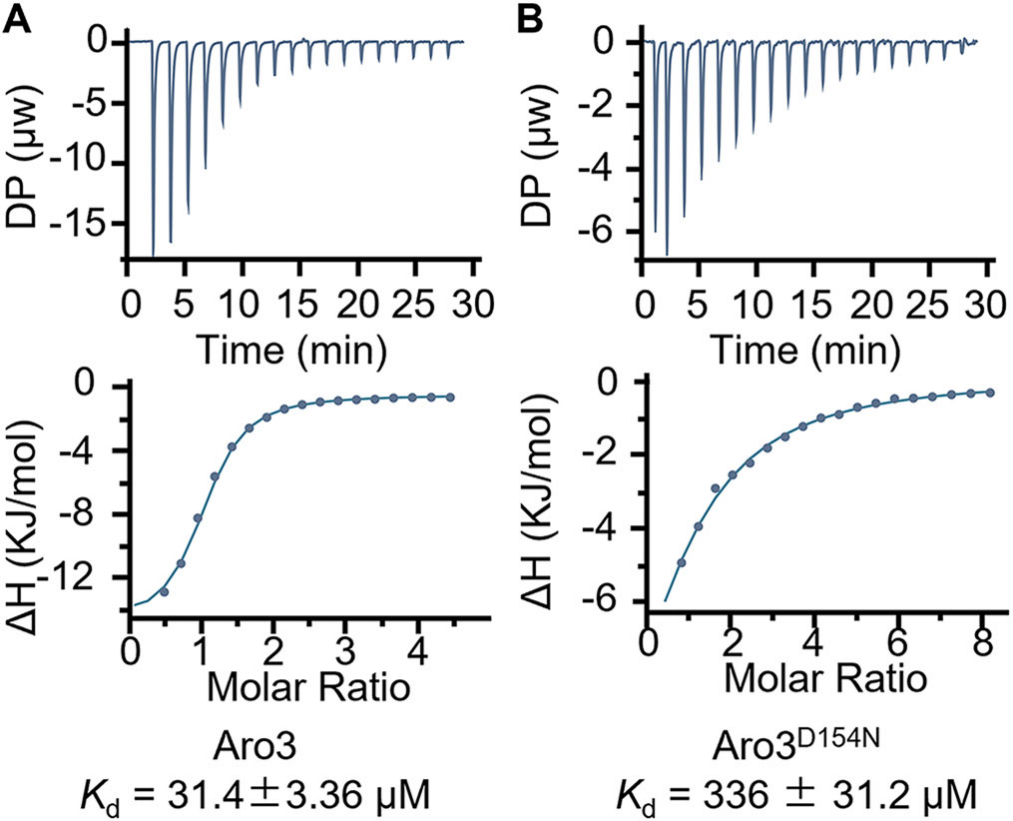
ITC analysis of _L_-Phe binding to Aro3 variants. **A**, **B** ITC titrations of _L_-Phe to the wild type of Aro3 (**A**) and Aro3^D154N^ (**B**). The upper panels present the binding isotherm and the lower panels show the integrated heat for per mole of injectant as a function of molar ratio.

We next employed isothermal titration calorimetry (ITC) to quantitatively characterize the interactions between Aro3 and Aro3^D154N^ with _L_-Phe. The two recombinant Aro3 forms showed enthalpy-driven binding of _L_-Phe with physiologically relevant *K*_d_ (Fig. 5). Among them, the wild-type enzyme demonstrated high binding affinity for _L_-Phe with a *K*_d_ of 31.4 μM. The mutant Aro3^D154N^ exhibited weaker interactions and higher *K*_d_ value, 10-fold of the wild-type *K*_d_. The ITC measurements are consistent with the observation that D154N reduced the binding affinity for _L_-Phe in MD stimulations.

### The D to N mutation in other type Iα DAHPS enzymes

Sequence alignments of DAHPS enzymes revealed high conservativity of the Asp residue corresponding to the D154 of yeast Aro3 (Supplementary Figure 6). Considering that all type Iα DAHPS enzymes with known structures share highly similar overall folding and domain architectures^5^, we decided to test the effects of substituting this residue with Asn in other enzymes of this family. We started the investigation of Iα DAHPS from commonly used model organisms known to be inhibited by _L_-Tyr instead of _L_-Phe, including Aro4 from *S. cerevisiae* and AroF from *E. coli*. We constructed Aro4^D161N^ and AroF^D147N^ and examined them in the established plate assays. Overexpression of both mutants recovered growth inhibited by _L_-Tyr (Supplementary Figure 7). Strikingly, Aro4^D161N^ was found as effective as Aro4^K229L^ (Supplementary Figure 7) that is widely used to unlock carbon flux into the AAAs biosynthetic pathway in yeast^1^.

**Fig. 6:**
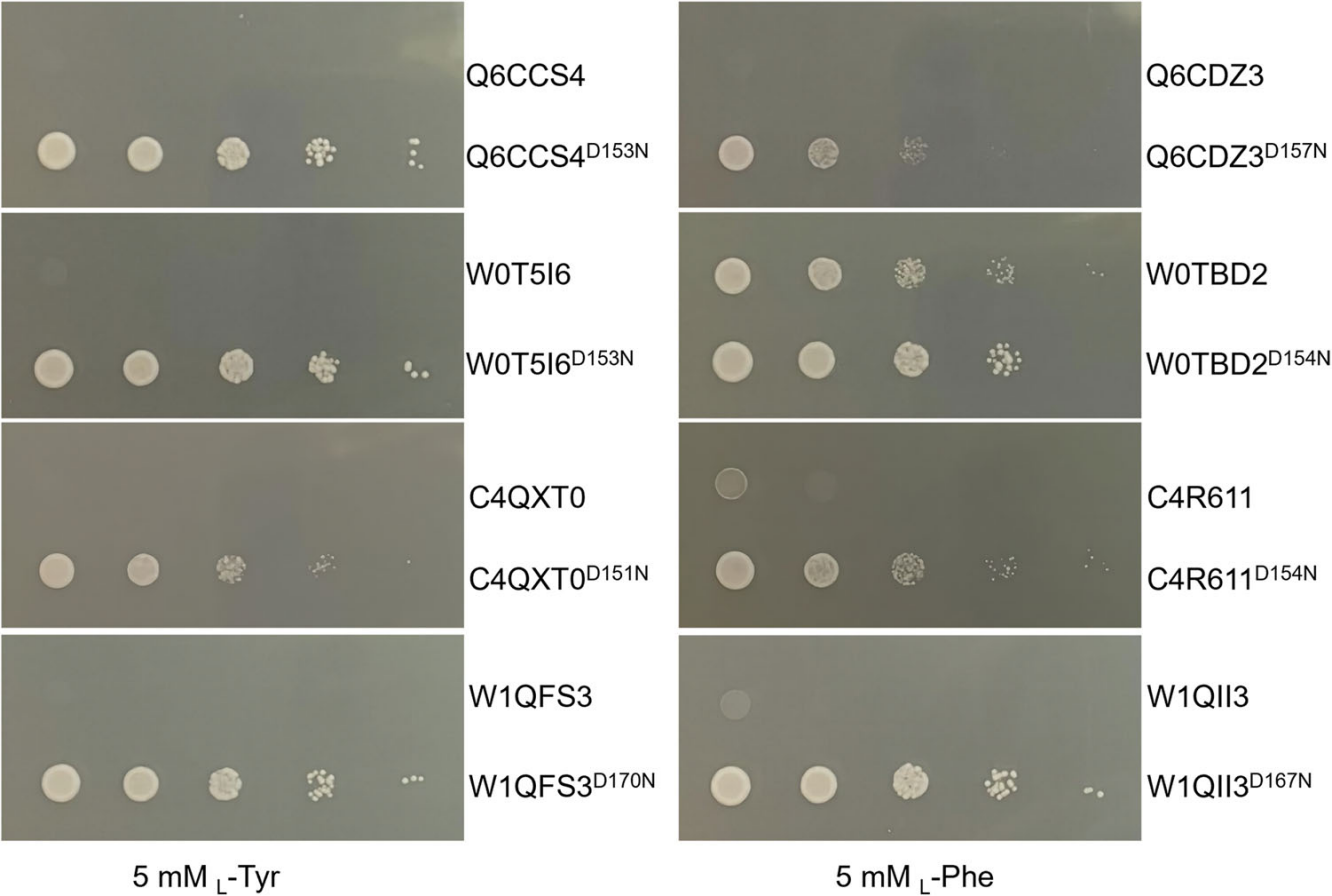
Growth inhibition by _L_-Phe or _L_-Tyr of yeast strains carrying various Type Iα DAHPS enzymes from non-conventional industrial hosts. The restoration of *TRP1* gene and the exchange of the *ARO3* promoter with the constitutive *TEF1* promoter were performed in CEN.PK2-1C *Δaro4* strain, then the genes encoding various Type Iα DAHPS enzymes were integrated into the *ARO3* locus. Q6CCS4 and Q6CDZ3 were encoded by *YlARO4 and YlARO3* from *Yarrowia lipolytica*; C4QXT0 and C4R611 were encoded by *KpARO4* and *KpARO3* from *Komagataella phaffii* GS115 (*Pichia pastoris*); W0T5I6 and W0TBD2 were encoded by *KmARO4* and *KmARO3* from *Kluyveromyces marxianus* strain DMKU3-1042 (*Candida kefyr*); W1QFS3 and W1QII3 were encoded by *OpARO4* and *OpARO3* from *Ogataea parapolymorpha* strain ATCC 26012 (*Hansenula polymorpha*), respectively. Mutants of these Type Iα DAHPS enzymes were designed with the analogous D154N of Aro3. The yeast strains were cultivated in MV liquid medium, harvested at 36 h, diluted at OD_600_ = 1.0, and serially 10-fold diluted to spot onto the MV solid medium in the presence of 5 mM _L_-Phe or _L_-Tyr. Plates were incubated at 30 °C for 60 h.

We next expanded our studies with focuses on type Iα DAHPS enzymes from various organisms that are not conventionally used in industry, including Q6CCS4 and Q6CDZ3 from *Yarrowia lipolytica*, C4QXT0 and C4R611 from *Komagataella phaffii* GS115 (*Pichia pastoris*), W0T5I6 and W0TBD2 from *Kluyveromyces marxianus* strain DMKU3-1042 (*Candida kefyr*), W1QFS3 and W1QII3 from *Ogataea parapolymorpha* strain ATCC 26012 (*Hansenula polymorpha*). Some unique properties of these microbial organisms were recently appreciated and they started being used as microbial cell factories^21–24^. However, lack of knowledge about their type Iα DAHPS enzymes and methods to bypass the allosteric inhibition impedes boosting carbon flux into the shikimate pathway for the high yield production of aromatic compounds. According to previous studies, among all our tested enzymes, Q6CDZ3, C4R611, W0TBD2, and W1QII3 contain a serine residue corresponding to the Ser219 of yeast Aro3 and are likely sensitive to _L_-Phe, while a glycine residue at the same position in Q6CCS4, C4QXT0, W0T5I6, and W1QFS3 may confer their sensitivity to _L_-Tyr inhibition^6,19,20^. Our plate assays with engineered yeast strains carrying the WT forms of these DAHPS enzymes confirmed such inhibitor specificities (Supplementary Figure 8). We next tested the D to N mutant forms of these enzymes, despite the differences of the inhibitors and the origins of the enzymes, a D to N single amino acid residue substitution demonstrated strong efficacy in relieving the growth inhibition by the enzyme inhibitor (Fig. 6).

### Metabolomic studies of the *ARO3*^*D154N*^ overexpressing strain

**Fig. 7:**
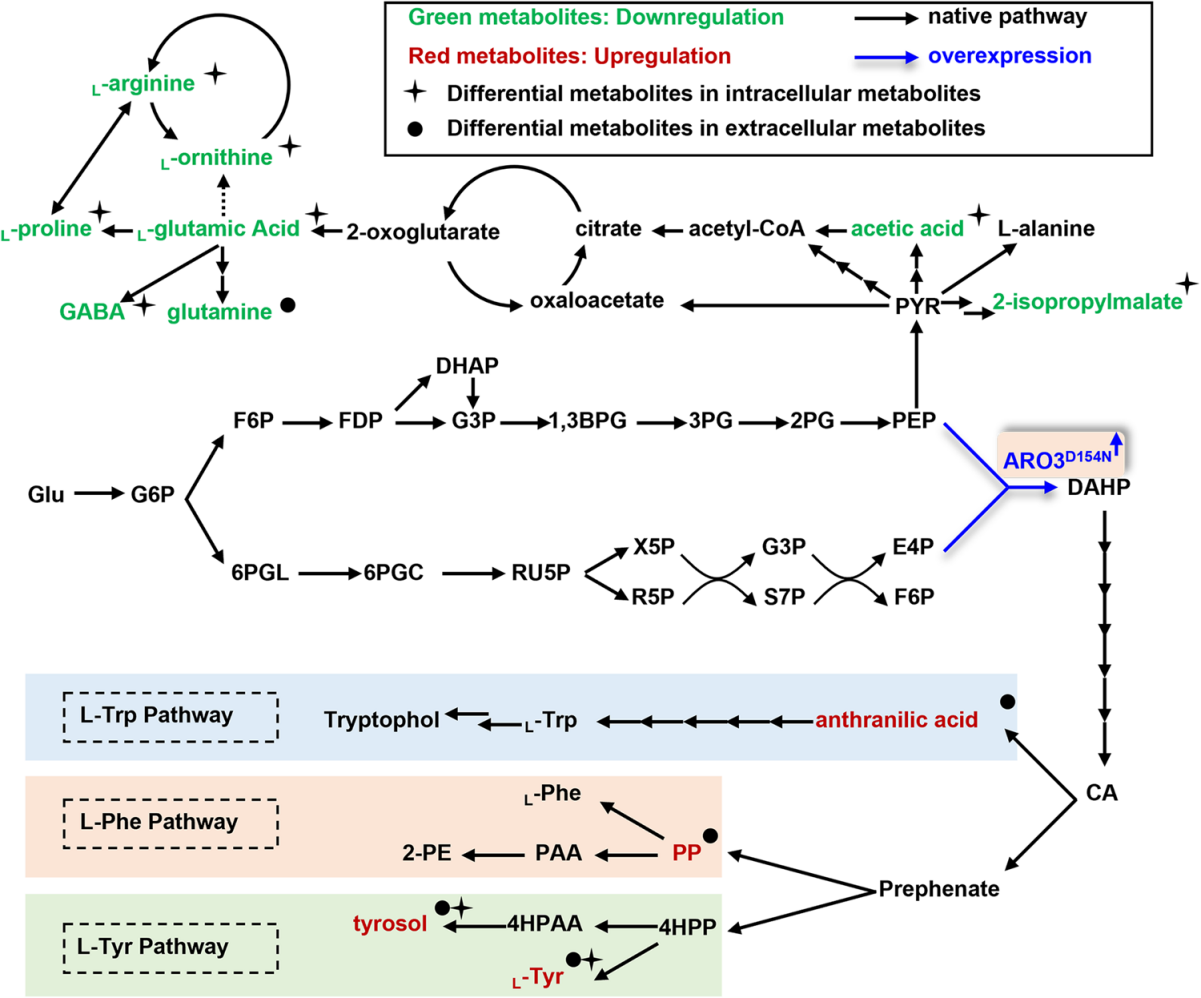
Differential metabolites between the wild-type strain and the *ARO3*^*D154N*^ overexpressing yeast strain. Orthogonal partial least squares discriminate analysis (OPLS-DA) was applied to figure out the differential metabolites between the groups. The downregulated metabolites were colored in green; the upregulated metabolites were in red and the metabolites with insignificant changes were in black. The intracellular and extracellular metabolites with significant differences in overexpressing *ARO3*^*D154N*^ yeast strains in comparison to the wild-type strains were marked with asterisk and dot, respectively. Black arrows represent the native pathways in *S. cerevisiae*; blue bold arrow represents the overexpressed gene in this study. Metabolite abbreviations: Glu, glucose; G6P, glucose 6-phosphate; F6P, fructose 6-phosphate; FDP, fructose 1,6-bisphosphate; G3P: D-glyceraldehyde 3-phosphate; DHAP, dihydroxyacetone phosphate; 1,3BPG, 1,3-bisphospho-D-glycerate; 3PG, 3-phospho-D-glycerate; 2PG, 2-phospho-D-glycerate; 6PGL, D-glucono-1,5-lactone 6-phosphate; 6PGC, 6-phospho-D-gluconate; RU5P: D-ribulose 5-phosphate; X5P: D-xylulose 5-phosphate; R5P: D-ribose 5-phosphate; PRPP, 5-phospho-alpha-D-ribose 1-diphosphate; S7P: sedoheptulose 7-phosphate; PEP: phosphoenolpyruvate; E4P: D-Erythrose 4-phosphate; DAHP: 3-deoxy-D-*arabino*-heptulosonate-7-phosphate; CA, chorismate; PP, phenylpyruvic acid; PAA, phenylacetaldehyde; 2-PE, 2-phenylethanol; _L_-Phe, _L_-phenylalanine; 4HPP, 4-hydroxyphenylpyruvic acid; 4HPAA, 4-hydroxyphenylacetaldehyde; _L_-Tyr, _L_-tyrosine; _L_-Trp, _L_-tryptophan; PYR, pyruvate; GABA, γ-aminobutyric acid.

Next, we performed metabolomic analyses on the *ARO3*^*D154N*^ overexpressing strain (strain A3_3) with the origin CEN.PK2-1C strain and wild-type ARO3 overexpressing strain as controls. We examined both intracellular and extracellular concentrations of AAAs derivatives including anthranilic acid, phenylpyruvic acid, _L_-tyrosine, and tyrosol. It was found that all of them increased significantly in the *ARO3*^*D154N*^ overexpressing strain but not in the wild-type *ARO3* overexpressing strain in comparison with the origin CEN.PK2-1C strain levels. By contrast, the concentrations of metabolites that branch out from PEP decreased in the *ARO3*^*D154N*^ overexpressing strain. These include intracellular 2-isopropylmalate, acetic acid, _L_-aspartic acid, GABA (γ- aminobutyric acid), _L_-proline, _L_-ornithine, and _L_-arginine, and extracellular glutamine (Fig. 7 and Supplementary Figures 9–10). The observation that metabolites of remarkably lowered concentrations are downstream products of the pyruvate metabolism in *S. cerevisiae* (Fig. 7), indicates that overexpression of *ARO3*^*D154N*^ shuttles carbon fluxes from pyruvate metabolism to the synthesis of AAAs.

### Effects of *ARO3*^*D154N*^ overexpression in tyrosol and salidroside overproducing engineered strains

**Fig. 8:**
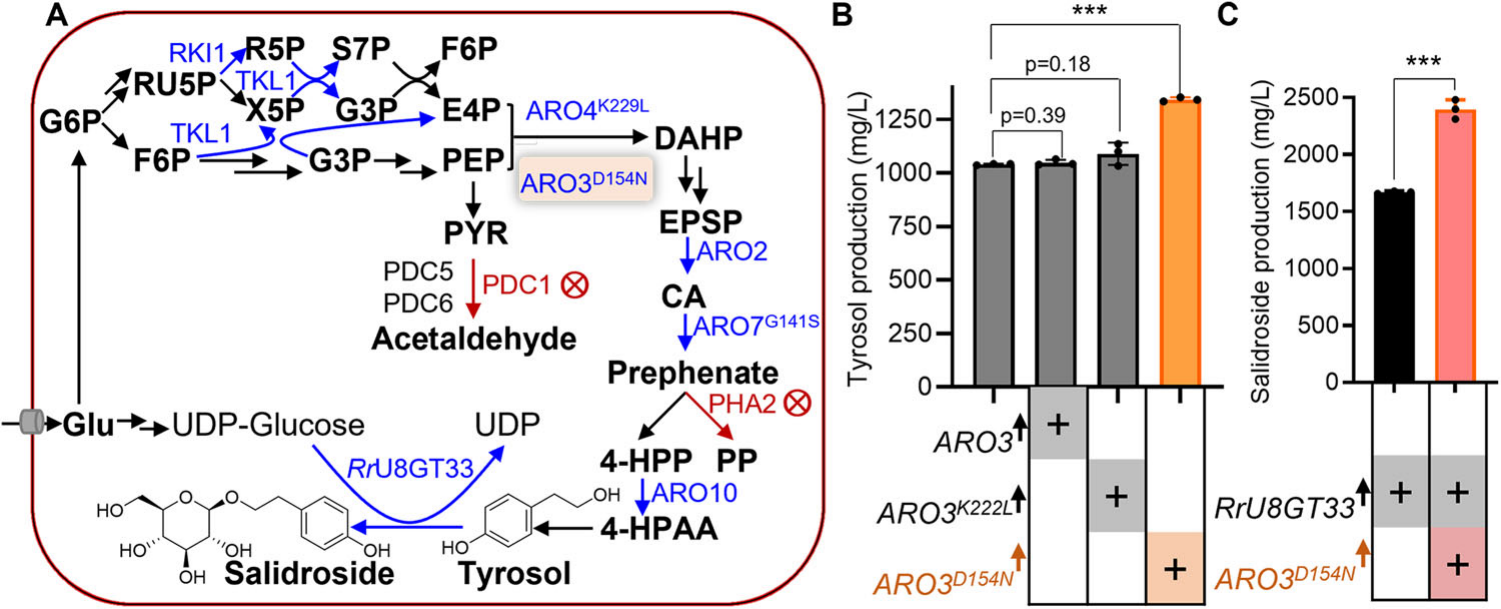
The overexpression of *ARO3*^*D154N*^ enhanced tyrosol and salidroside production in yeast strains. **A** The biosynthetic pathway of tyrosol and salidroside in *S. cerevisiae*. The pentose phosphate pathway, glycolysis, shikimate pathway, and _L_-tyrosine branch were systematically engineered to relieve the bottlenecks for synthesis of tyrosol^11, 27^. Blue and red arrows represent the gene overexpression and gene deletion, respectively. The overexpression of *ARO3*^*D154N*^ was used to divert carbon flux towards the formation of tyrosol and salidroside. Metabolite abbreviations: Glu, glucose; G6P, glucose-6-phosphate; RU5P, D-ribulose 5-phosphate; X5P, D-xylulose 5-phosphate; R5P, D-ribose 5-phosphate; S7P, sedoheptulose 7-phosphate; G3P, D-glyceraldehyde 3-phosphate; F6P, beta-D-Fructose 6-phosphate; PEP, phosphoenolpyruvate; E4P, D-erythrose 4-phosphate; PYR, pyruvate; DAHP: 3-deoxy-D-*arabino*-heptulosonate-7-phosphate; EPSP: 5-enolpyruvyl-3-shikimate; CA, chorismate; 4-HPP: 4-hydroxyphenylpyruvate; PP: phenylpyruvate; 4-HPAA: 4-hydroxyphenylacetylaldehyde. **B** The overexpression of *ARO3*^*D154N*^ enhanced the tyrosol production. **C** The overexpression of *ARO3*^*D154N*^ enhanced the salidroside production. Error bars represent the standard deviation of three biological replicates. Source data are available in Supplementary Data 4. Statistical analysis was performed by using Student’s t test (***p < 0.001).

Tyrosol and salidroside are widely used as ingredients of functional foods, cosmetics, and medicines owing to their anti-oxidative and many other properties beneficial to human health^25,26^. Historically, our laboratory constructed a yeast strain, namely TY4 with a high yield of tyrosol^27^ (Fig. 8A). We used TY4 as a chassis to overproduce salidroside based on our discovery that glycosylation of tyrosol to form salidroside is efficiently catalyzed by a glycosyltransferase from *Rhodiola rosea*, the *Rr*U8GT33^11^ (Fig. 8A). Integration of the overexpressing *ARO3*^*D154N*^ cassette into TY4 produces TY5, which results in a 29% increase of tyrosol titer, reaching record-high 1.3 g/L. By comparison, overexpression of the wild-type *ARO3* or *ARO3*^*K222L*^ did not show statistically significant effects (Fig. 8B). TY4 and TY5 were engineered to overexpress *Rr*U8GT33 as previously reported^11^, and compared with each other for salidroside production. Overexpression of *ARO3*^*D154N*^ was noticed to increase salidroside production by 44%, reaching 2.4 g/L, which is also the highest ever reported in flask fermentation (Fig. 8C). The products of tyrosol and salidroside was confirmed by LC-MS/MS and by comparing with standard compounds (Supplementary Figures 11–12).

## Discussion

The feedback inhibition-resistant mutant Aro4^K229L^ has been widely used in yeast engineered for aromatic compound overproduction^1^, whereas report of Aro3 allele for similar purposes was rare. Christine Brückner et al. showed that the overexpression of an analogous Aro3 mutant, Aro3^K222L^ increased the yield of protocatechuic acid^10^. It is intriguing that studies by others and the present work failed in observing any positive effect of Aro3^K222L^ mutant^11–13^. The discrepancy could have been due to usage of different yeast genetic backgrounds. We chose the *aro4* deleting strain in order to avoid interference by its specific inhibitor _L_-Tyr, while the Brückner group used a strain deletion of AroE that encodes the E-domain of Aro1 with dehydrogenase activity. Deletion of AroE hinders the carbon fluxes towards AAA biosynthetic pathway, thus decreases the intracellular concentration of _L_-Phe^10^. Perturbation by AroE deletion makes interpretation of the data difficult, given that _L_-Phe inhibition on Aro3 is dose-dependent. It was also noted that the Brückner group codon-optimized *ARO3*^*K222L*^, but not the control *ARO3* sequence as stated by themselves, which may have resulted in different expression levels of the two enzyme versions accounting for different yields of protocatechuic acid. Nevertheless, no positive effects of *ARO3*^*K222L*^ were observed in any of our in vivo systems, where genomic sequences were used in constructing the overexpression of both *ARO3* and its mutants to avoid potential variables of protein levels. Furthermore, our results of the in vitro enzyme activity assays using recombinant proteins allowing accurate quantitation should have been more conclusive.

The crystal structure of Aro3 and its modeled complex with Phe reported in this study allow comparison with the yeast Aro4 Tyr complex structure (PDB: 1OF6), which helps to interpret why *ARO4*^*K229L*^ relieves feedback inhibition and *ARO3*^*K222L*^ doesn’t^6,18^. Tyr differs from Phe by its 4-OH group. Despite high similarity of inhibitor-enzyme interactions in both complexes, hydrogen bonding between the 4-OH group of Tyr and the backbone carbonyl group of T162 in Aro4 is absent in the Phe-Aro3 complex (Supplementary Figure 13). This interaction acts in concert with the K229L mutation to position Tyr in proximity to helices α3 (residues 164–159) and α3/β3 loop (residues 157–163) abolishing its inhibitory effect on Aro4. By contrast, Aro3^K222L^ alone doesn’t mobilize Phe in the pocket and thus Phe remains inhibitory.

Although the D154 of Aro3 and its counterparts of other type Iα DHAPS are located outside the inhibitor binding cavity^14,20,28^, substitution by Asn weakened interactions between Q159/K222 and the enzyme inhibitor (Fig. 4). Type Iα DAHPS enzymes share highly similar overall folding and domain architectures, and the D154 of Aro3 was highly conserved in type Iα DAHPS enzymes through multiple sequence alignment (MSA) using the ConSurf Server (https://consurf.tau.ac.il/consurf_index.php). The modeled complex structure and MD simulation taken together suggest that the D to N mutation may endow all type Iα DHAPS enzymes with advantages for evading feedback inhibition in fermenting industry for aromatic compounds. This was supported by our preliminary experiments using the yeast plate assay. The origins of tested DHAPS include *Yarrowia lipolytica, Pichia pastoris*, *Kluyveromyces marxianus*, and *Hansenula polymorpha*, each having uniquely attractive properties, high acetyl-CoA contents, high protein production, thermotolerance, and competency in protein glycosylation respectively^21–24^. Therefore, these microbial organisms started being used as cell factories for various chemical compounds including aromatic products. High levels of resveratrol, scutellarin, and _L_-tyrosine derivatives have recently been produced by engineered *Yarrowia lipolytica*^29,30^ and *Pichia pastoris* strains^31^. In most of these studies, Aro4^K229L^ from *S. cerevisiae* was introduced into the non-conventional hosts to facilitate carbon fluxes towards AAAs synthetic pathway^23,29–32^. Our yeast plate assays were initial attempts to understand the DHAPS from these organisms, which may shed light on further increase of aromatic compound production in non-conventional platforms using their native enzymes. The potential of this feedback-unlocked strategy in industrial hosts was fully displayed by the record-high titers of tyrosol and salidroside in *S. cerevisiae* strains overexpressing *ARO3*^*D154N*^.

## Methods

### Protein expression and purification

The mutants of *ARO3* were constructed by one-step site-directed mutagenesis method^33^ and cloned to pET21 via Gibson cloning^34^, containing a His_6_ tag at the C-terminus. All proteins in this study were expressed in *E. coil* strain Rosetta 2 (DE3). The *E. coil* cells were cultured at 37 °C in LB medium (containing 100 mg/L ampicillin) until OD_600_ reached 0.8, and then induced by 0.2 mM isopropyl β-D-thiogalactopyranoside (IPTG) at 16 °C for 18–20 h. Cells were harvested by centrifugation at 4000 × *g* for 12 min, re-suspended in the lysis buffer (20 mM Tris-HCl buffer, pH 8.0, 150 mM NaCl, 10% glycerol), and lysed using a high-pressure French press (Antos Nano Technology Co., Ltd., China). After centrifugation, the supernatant was loaded onto a Nickel-charged nitrilotriacetic acid (NTA) column and the column was washed with the wash buffer (20 mM Tris-HCl buffer, pH 8.0, 150 mM NaCl, 10% glycerol, 10 mM imidazole), and then eluted with the elution buffer (20 mM Tris-HCl buffer, pH 8.0, 150 mM NaCl, 10% glycerol, 250 mM imidazole). The proteins were further purified by gel filtration chromatography (Superdex 200 Increase 10/300 GL, GE Healthcare) with the storage buffer (50 mM potassium phosphate buffer, pH 6.5, 150 mM NaCl). The purified proteins were stored at −80 °C after flash-frozen in liquid nitrogen until use.

### Crystallization and structure determination

Crystals were grown using the sitting drop vapor diffusion method at 18 °C by mixing equal volumes (1.5 μL + 1.5 μL) of protein solution (8 mg/mL) and crystallization buffer (0.1 M BICINE, pH 9.0, 0.1 M sodium chloride, 20% v/v polyethylene glycol monomethyl ether 550). The data sets were collected at beamline BL19U1 of Shanghai Synchrotron Radiation Facility (SSRF) and processed with the XDS using Aimless to scale in the CCP4 suite. The structure was solved by molecular replacement using the CCP4 suite with the structure model that was predicted by AlphaFold2^35^ as a template. The final model was refined through multiple cycles of building and refinement and the R_work_/R_free_ values were 25.2%/29.5%, respectively (Table 1). The protein crystal data is provided with Supplementary Data 1.

### Modeling and molecular dynamics simulations

The structural model of Aro3 with _L_-Phe complex was constructed using AutoDockTools-1.5.6 program. The box was set relative to the binding pocket in the known DAHPS structure complexed with _L_-Phe (PDB: 1KFL and 4UC5), using the following parameters: grid box center, x, y, z = −14.959, −35.752, 13.189; grid box size in x, y, z direction = 20, 20, 30). Other parameters were used as default. Additionally, the selection of the binding mode for further analysis was also based on referencing the known structures (PDB: 1KFL and 4UC5). The molecular dynamics simulations (MD) were built by CHARMM-GUI and run through using Gromacs with the CHARMM36 force field (http://www.gromacs.org/)^36^. The protein was placed into TIP3P water and 0.15 M NaCl (in addition to the counterions used to neutralize charge), and the box size was 117 × 117 × 117 Å^3^ with periodic boundary conditions. The system was energy minimized using the steepest descents method over 5000 steps. Next, the system was relaxed by applying restraints using the standard CHARMM-GUI equilibration protocol. The simulation was performed for 1000 ns without positional restraints with 2 fs time steps at temperature of 303 K and constant pressure (1 bar). The structural coordinates of the initial and final conformation obtained from our simulations are provided with Supplementary Data 2. During the MD process, the LINCS algorithm was used to constrain the bond length^37^. The smoothed cutoff distance for non-bonded interactions was set to 12 Å and long-range electrostatic interactions were computed with the Particle Mesh Ewald (PME) method^38^.

### Isothermal titration calorimetry

For isothermal titration calorimetry (ITC measurement), _L_-Phe was dissolved in the storage buffer (50 mM potassium phosphate buffer, pH 6.5, 150 mM NaCl). Isothermal titration calorimetry was carried out at 25 °C on an ITC200 system (MicroCal, UK). 8 mM _L_-Phe was titrated into 200 μM Aro3 protein in storage buffer. A titration of _L_-Phe into storage buffer was carried out as a control. Data were fitted and analyzed using MicroCal PEAQ-ITC Analysis Software (Malvern Panalytical, UK).

### Enzyme assays

The reaction mixture (250 μL) contained 100 mM potassium phosphate buffer (pH 6.5), 0.5 mM phophoenolpyruvate (PEP) and 0.5 mM erythrose-4-phosphate (E4P). To check the allosteric control of Aro3 variants, either 0.1 mM, 0.5 mM, 1 mM, 2.5 mM, 5 mM, or 10 mM of _L_-Phe (final concertation) was added to the reaction mixture. The reaction was initiated by the addition of Aro3 protein (2 μg) and was incubated at 30 °C for 5 min. Then the DAHP was detected via the method described by Sprinson^39^ with some modifications. Firstly, 100 μL of 10% w/v trichloroacetic acid solution was added into the mixture to stop the reaction. Next, the supernatant was obtained by centrifugation at 12,000 × *g* for 5 min and 125 μL periodic acid (25 mM in 0.125 M H_2_SO_4_) was added to the supernatant and the mixture was incubated at 25 °C for 45 min. To eliminate excess periodate, 250 μL of 2% (w/v) sodium arsenite in 0.5 M HCl was added into the mixture. After incubation at room temperature for 2 min, 1 mL of 0.3% (w/v) 2-thiobarbituric acid (pH 2.0) was added to the tube and then the tube was heated at 100 °C for 5 min. The pink color developed was rapidly measured at 549 nm by using SynergyMx Multi-Mode Microplate Reader (BioTek, USA).

### Strain construction

*S. cerevisiae* strains are listed in Supplementary Table 1. The plasmids used in this study were listed in Supplementary Table 2 and the primers were listed in Supplementary Table 3. The endogenous genes, promoters and terminators of *S. cerevisiae* were amplified from genomic DNA of CEN.PK2-1C. The DNA sequences of AroF, *YlARO4*/*YlARO3*, and *KpARO4*/*KpARO3* were cloned form the genomic DNA of *E. coli*, *Yarrowia lipolytica*, and *Komagataella phaffii* GS115, respectively. The genes of *KmARO4*/*KmARO3* and *OpARO4*/*OpARO3* were synthesized by GENEWIZ, China. All the DAHPS sequences were listed at Supplementary Data 3.

Plasmids pTY1-3 were constructed in our previous study^11^. To construct plasmids pTY4, the plasmid pRS405 was firstly linearized using restriction enzymes *HindIII*-HF and *BamHI*-HF (New England Biolabs, US). Then the *TEF1* promoter, *ARO3* fragment and *PGK1* terminator were ligated together through overlap extension PCR and ligated to the linearized pRS405. Plasmids pTY5 and pTY6 harboring *ARO3*^*K222L*^ and *ARO3*^*D154N*^ respectively were constructed through the similar procedure. Plasmids pTY4-6 were linearized at their auxotrophic *LEU2* marker and integrated at the *LEU2* site of yeast. Yeast transformation was carried out through the LiAc/ssDNA/PEG method^40^. The corrected yeast clones were selected on SC-LEU medium. The CRISPR-Cas9 system was applied to modify the genome, which was similar in our previous process^11^.

### Culture conditions

Yeast strains were grown on YPD medium (20 g/L peptone, 10 g/L yeast extract and 20 g/L glucose). The yeast transformants were screened on YPD containing 200 μg/mL G418 or synthetic complete drop-out medium. DH5α and DH10β were used for sub-cloning and Rosetta2 (DE3) was used for protein expression. The *E. coli* strains were cultivated in LB medium with 100 μg/mL ampicillin or 50 μg/mL kanamycin at 37 °C. The MV medium (0.15% yeast nitrogen base, 0.52% ammonium sulfate, 2% glucose, 1% succinic acid, 0.3% KOH (pH 4.0 for liquid medium) or 8.5% KOH (pH = 5.5 for solid medium) was supplemented with histidine, leucine, and uracil to test the feedback sensitivity of _L_-Phe on Aro3 variants. Yeast strains were cultured in MV liquid medium 36 h, diluted at OD_600_ = 1.0, and then were serially 10-fold diluted to spot onto the MV solid medium in the presence of different concentrations of _L_-Phe, _L_-Trp, or _L_-Tyr. Plates were incubated at 30 °C for 60 h.

### Metabolomics analysis

Intracellular and extracellular metabolites were analyzed by Metware Biotech Co., Ltd (Wuhan, China). In brief, yeasts were grown on YPD medium at 30 °C for 48 h, and the cultivated yeasts were centrifuged at 5000 rpm for 5 min. The harvested cells and the supernatant were sent to Metware Biotech Co., Ltd (Wuhan, China) for further analysis. The harvested cells were washed with Milli-Q water and extracted for intracellular metabolites. The supernatant was used for extracellular metabolites analysis. The metabolites were extracted by adding three times of ice-cold methanol and qualitatively analyzed using a LC-MS/MS system. The analytical conditions were as follows, UPLC column, Waters ACQUITY UPLC HSS T3 C18 (1.8 µm, 2.1 mm × 100 mm); column temperature, 40 °C; flow rate, 0.4 mL/min; injection volume, 2 μL; solvent system, water (0.1% formic acid): acetonitrile (0.1% formic acid); gradient program, 95:5 V/V at 0 min, 10:90 V/V at 11.0 min, 10:90 V/V at 12.0 min, 95:5 V/V at 12.1 min, 95:5 V/V at 14.0 min. Each biological sample was measured in triplicates.

### Fermentation and analysis of products

Yeast strains were picked from pre-cultured plates and cultured in 4 mL YPD medium at 30 °C, shaking at 220 rpm for 24 h. Portions of pre-cultured mixture were then transferred into 50 mL YPD medium in 250 mL shake flasks until the OD_600_ reached ~0.2. The strains were cultured at 30 °C, shaking at 220 rpm for 84 h. 1 mL of the culture was collected and centrifuged at 5000 rpm for 5 min. The supernatant was stored at −20 °C until analysis.

Tyrosol, salidroside, tryptophol, and 2-phenylethanol were analyzed using an Agilent HPLC 1260 series instrument equipped with a Zorbax SB-C18 column (Agilent, 5 μm, 4.6 mm × 250 mm). The injection volume was 10 μL, and samples were maintained at 30 °C. A diode array detector (DAD) was used for the analysis (identification and quantification) of the compounds. Mobile phase A was 0.05% formic acid in water and solvent B was acetonitrile, and phase A and phase B were used for following analysis. The flow rate was set to 1.0 mL/min. Tyrosol and salidroside were analyzed by using a gradient method with two solvents: 8% B for 18 min, 5% B for 2 min, 5–95% B in 10 min, 95% B for 5 min, and 8% B for 5 min. The absorbance was set at 224 nm. For the analysis of 2-phenylethanol, the profile was as follows: 40% B for 20 min, 5% B for 5 min, 5–95% B in 5 min, and finally return to 40% B for 10 min. The absorbance of 2-phenylethanol was set at 210 nm. For the analysis of tryptophol, samples were analyzed for 50 min using the following gradient method: 8% B for 18 min, 25% B for 16 min, 95% B for 4 min, and 8% B for 10 min. The absorbance of tryptophol was set at 210 nm. The products were analyzed using an LC-ESI-MS/MS system (Nexera UHPLC LC-30A and AB SCIEX Triple Quad 5500 system). The Hypersil GOLD C18 column (1.9 μm, 100 mm × 2.1 mm; Thermo Fisher Scientific, USA) were used to separate tyrosol and salidroside in samples. The samples were maintained at 35 °C. The flow rate was 0.4 mL/min, and the injection volume was 1.0 μL. The mobile phases were 0.1% formic acid in water (A) and acetonitrile (B). The mobile phase for the produccts analysis was 2% B, which was applied for 5.0 min. For the MS2 model, the daughter ion of tyrosol (*m*/*z*, 137.1) and salidroside (*m*/*z*, 299.1) was scanned for in the range 50–200, 50–350, respectively.

### Reporting summary

Further information on research design is available in the Nature Portfolio Reporting Summary linked to this article.

## Supplementary information

Supplementary Information

Description of Additional Supplementary Files

Supplementary Data 1

Supplementary Data 2

Supplementary Data 3

Supplementary Data 4

Reporting Summary

## Data Availability

The atomic coordinates and structure factors have been deposited in the Protein Data Bank (https://www.wwpdb.org/) with PDB ID code 7YKC. The protein crystal data were also provided with Supplementary Data 1. The structural coordinates of the initial conformation and every 100 ns conformation obtained from our simulations are provided with Supplementary Data 2. Gene sequences were listed at Supplementary Data 3. Source data are available in Supplementary Data 4.
