# Supplementary material for: Mechanistic investigation of a D to N mutation in DAHP synthase that dictates carbon flux into the shikimate pathway in yeast
Source: Commun Chem. 2023 Jul 15;6:152. doi: 10.1038/s42004-023-00946-x (PMC10349828; doi:10.1038/s42004-023-00946-x)
Supplement: Supplementary file 1 — Supplementary Information [file 42004_2023_946_MOESM1_ESM.pdf]

Supplementary Data

Mechanistic investigation of a D to N mutation in DAHP synthase that dictates carbon flux into the shikimate pathway in yeast

Huayi Liu[1,2], Qingjie Xiao[3], Xinxin Wu[1], He Ma[1], Jian Li[1], Xufan Guo[1], Zhenyu Liu[1], Yan Zhang[4], Yunzi Luo[1.2*]

1. Frontiers Science Center of Synthetic Biology and Key Laboratory of Systems Bioengineering (Ministry of Education), School of Chemical Engineering and Technology, Tianjin University, Tianjin 300072, China
2. Georgia Tech Shenzhen Institute, Tianjin University, Tangxing Road 133, Nanshan District, Shenzhen, 518071 China
3. National Facility for Protein Science in Shanghai, Shanghai Advanced Research Institute (Zhangjiang Laboratory), Chinese Academy of Sciences, Shanghai 201210, China.
4. Tianjin Key Laboratory for Modern Drug Delivery & High-Efficiency, Collaborative Innovation Center of Chemical Science and Engineering, School of Pharmaceutical Science and Technology, Tianjin University, Tianjin 300072, China

*Address correspondence to Y.L (yunzi.luo@tju.edu.cn).

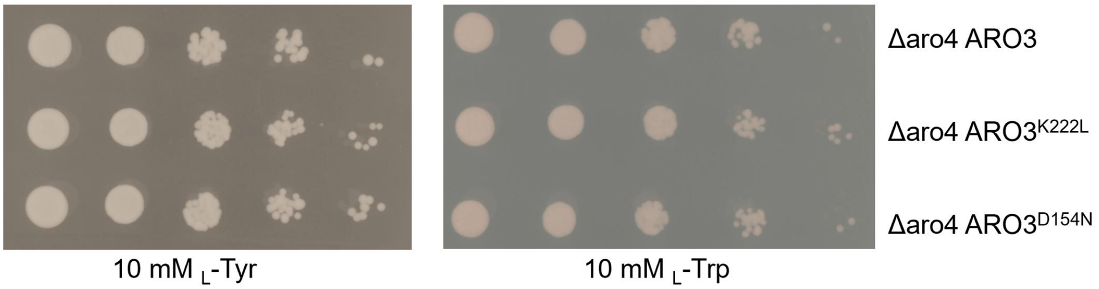

Supplementary Figure 1. Growth of yeast strains carrying Aro3 mutants. To compare the effects of different of Aro3 mutants, the restoration of *TRP1* gene and the exchange of the *ARO3* promoter with the constitutive *TEF1* promoter were performed in CEN.PK2-1C *Δaro4* strain, then the *in situ* mutations (*ARO3*^{K222L} and *ARO3*^{D154N}) were constructed. The yeast strains were cultivated in MV liquid medium, harvested at 36 h, diluted at OD_{600} = 1.0, and serially 10-fold diluted to spot onto the MV solid medium in the presence or absence of 10 mM L-Tyr or L-Trp. Plates were incubated at 30℃ for 60 hours.

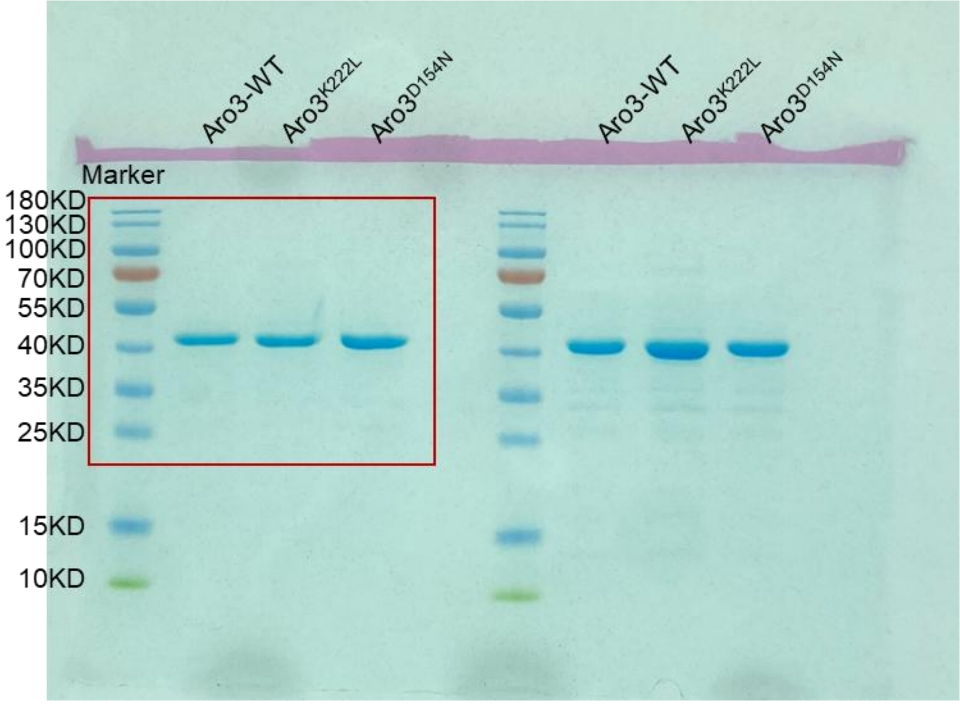

Supplementary Figure 2. The SDS-PAGE evaluation of recombinant proteins of Aro3, Aro3^{K222L} and Aro3^{D154N}. The cropped sections used in Figure 3 of this manuscript are indicated by red rectangles.

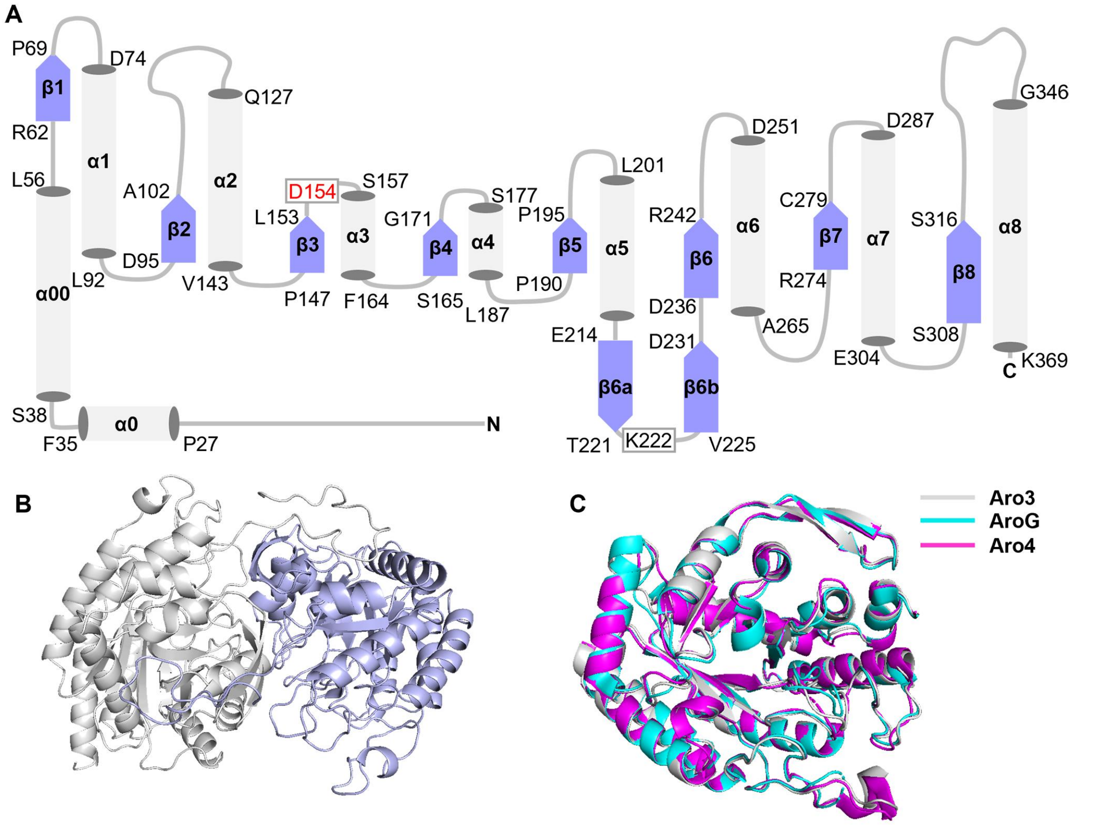

Supplementary Figure 3. The crystal structure of Aro3. (A) Topology plot of Aro3 from *S. cerevisiae*. The β-strands and α-helices of the central β/α barrel are shown in blue and light gray, respectively. Loops are shown in dark gray. The mutation D154N is highlighted in red. (B) The crystal structure of dimeric Aro3. (C) Comparison of the monomeric subunit of Aro3 (shown in gray, PDB: 7YKC) and that of AroG (shown in cyan, PDB: 1QR7) and that of Aro4 (shown in magenta, PDB: 1HFB).

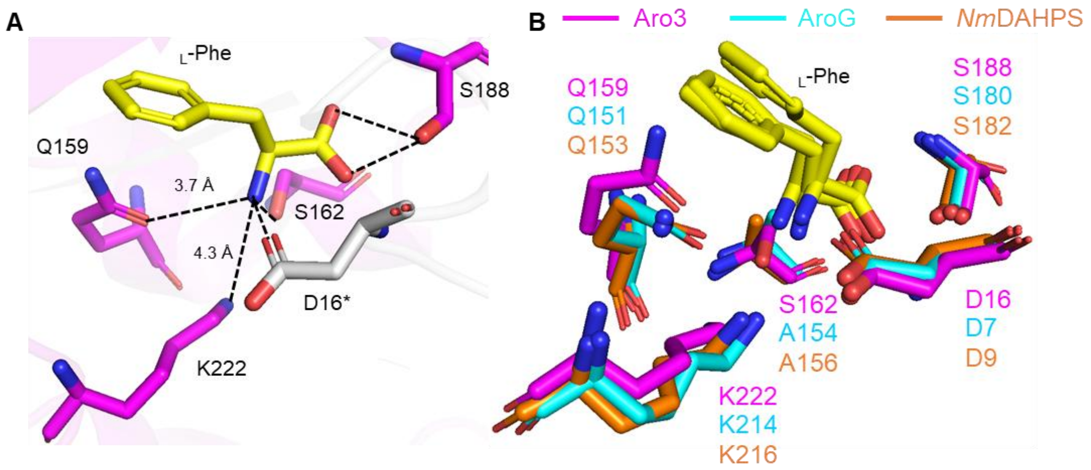

Supplementary Figure 4. Molecular docking with L-Phe into the binding pocket of Aro3. (A) Zoomed-in view of the distances between Q159/K222 and L-Phe of Aro3-Phe. L-Phe and the side chains of the binding residues are shown as sticks. (B) Superposition of the putative Phe binding residues of Aro3 (pink), AroG (PDB: 1KFL, cyan) and *Nm*DAHPS (PDB: 4UC5, orange).

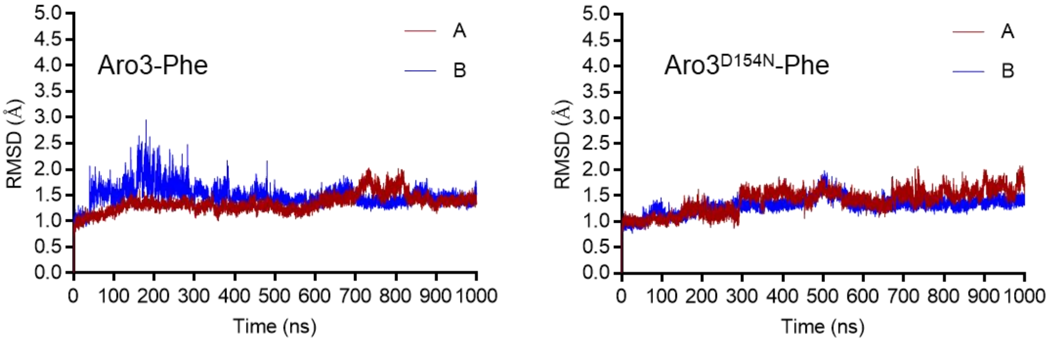

Supplementary Figure 5. The root-mean-square deviations (RMSD) values for the α-carbons of the dimer during the MD simulation. The root-mean-square deviations (RMSD) of the α-carbons was calculated using 15-371 residues because of the high flexibility of the N-terminal (1-14) tail.

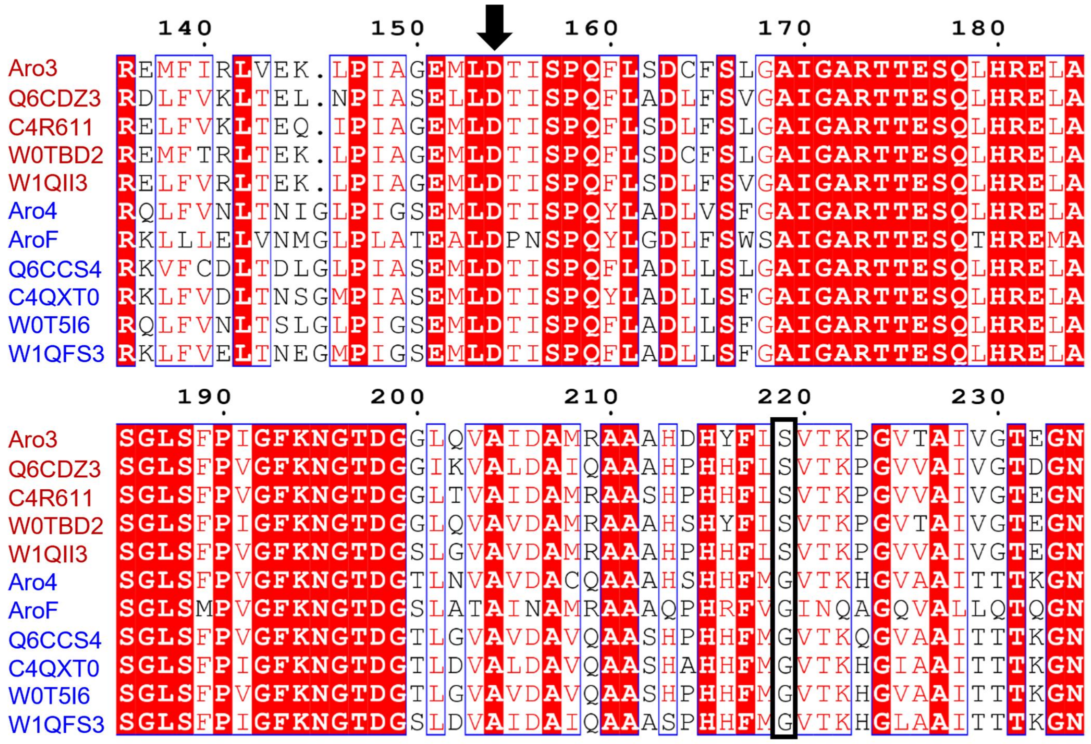

Supplementary Figure 6. Amino acid sequence alignment of Aro3 with several other Type Iα DAHP enzymes from non-conventional industrial hosts. Identical amino acids are in white font boxed in red. The conserved aspartic acid residue at the position 154 of yeast Aro3 are marked with an arrow. Residues identified (serine or glycine) as key residues in the recognition of the inhibitor were indicated in a black frame.

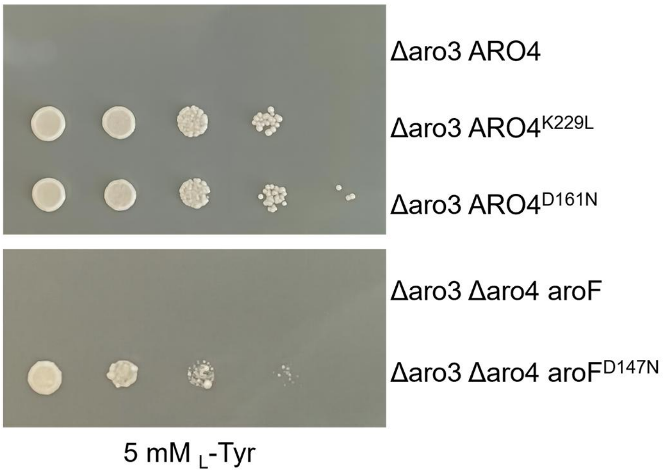

Supplementary Figure 7. Growth of yeast strains carrying Aro4, AroF and their mutants. The restoration of *TRP1* gene and the exchange of the *ARO3* promoter with the constitutive *TEF1* promoter were performed in CEN.PK2-1C *Δaro4* strain, then the *ARO4*^{D161N} and *AROF*^{D147N} genes were integrated into the *ARO3* locus. The yeast strains were cultivated in MV liquid medium, harvested at 36 h, diluted at OD_{600} = 1.0, and serially 10-fold diluted to spot onto the MV solid medium in the presence or absence of 5 mM L-Tyr. Plates were incubated at 30℃ for 60 hours.

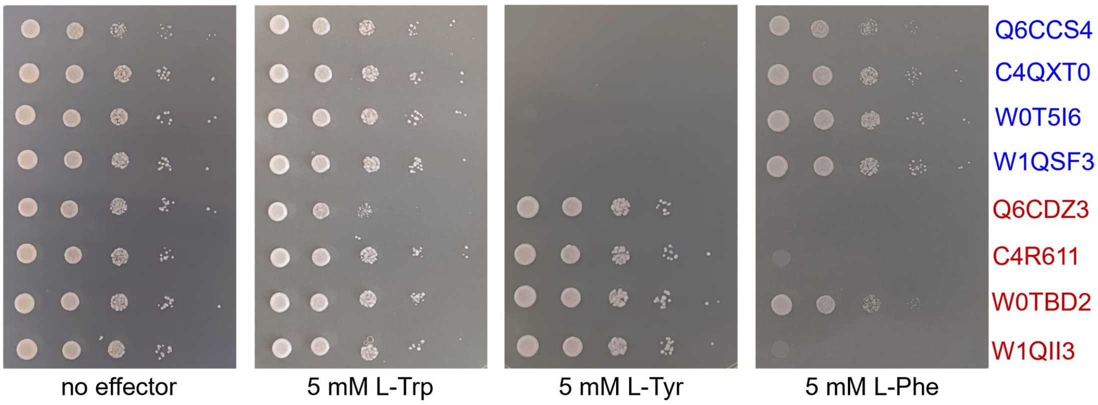

Supplementary Figure 8. Growth of yeast strains carrying various Type Iα DAHP enzymes. The restoration of *TRP1* gene and the exchange of the *ARO3* promoter with the constitutive *TEF1* promoter were performed in CEN.PK2-1C *Δaro4* strain, then the genes encoding various Type Iα DAHP enzymes were integrated into the *ARO3* locus. Q6CCS4 and Q6CDZ3 were encoded by *YlARO4 and YlARO3* from *Yarrowia lipolytica*; C4QXT0 and C4R611 were encoded by *KpARO4* and *KpARO3* from *Komagataella phaffii* GS115 (*Pichia pastoris*); W0T5I6 and W0TBD2 were encoded by *KmARO4* and *KmARO3* from *Kluyveromyces marxianus* strain DMKU3-1042 (*Candida kefyr*); W1QFS3 and W1QII3 were encoded by *OpARO4* and *OpARO3* from *Ogataea parapolymorpha* strain ATCC 26012 (*Hansenula polymorpha*), respectively. The yeast strains were cultivated in MV liquid medium, harvested at 36 h, diluted at OD_{600} = 1.0, and serially 10-fold diluted to spot onto the MV solid medium in the presence or absence of 5 mM L-Tyr, L-Trp, and L-Phe. Plates were incubated at 30℃ for 60 hours.

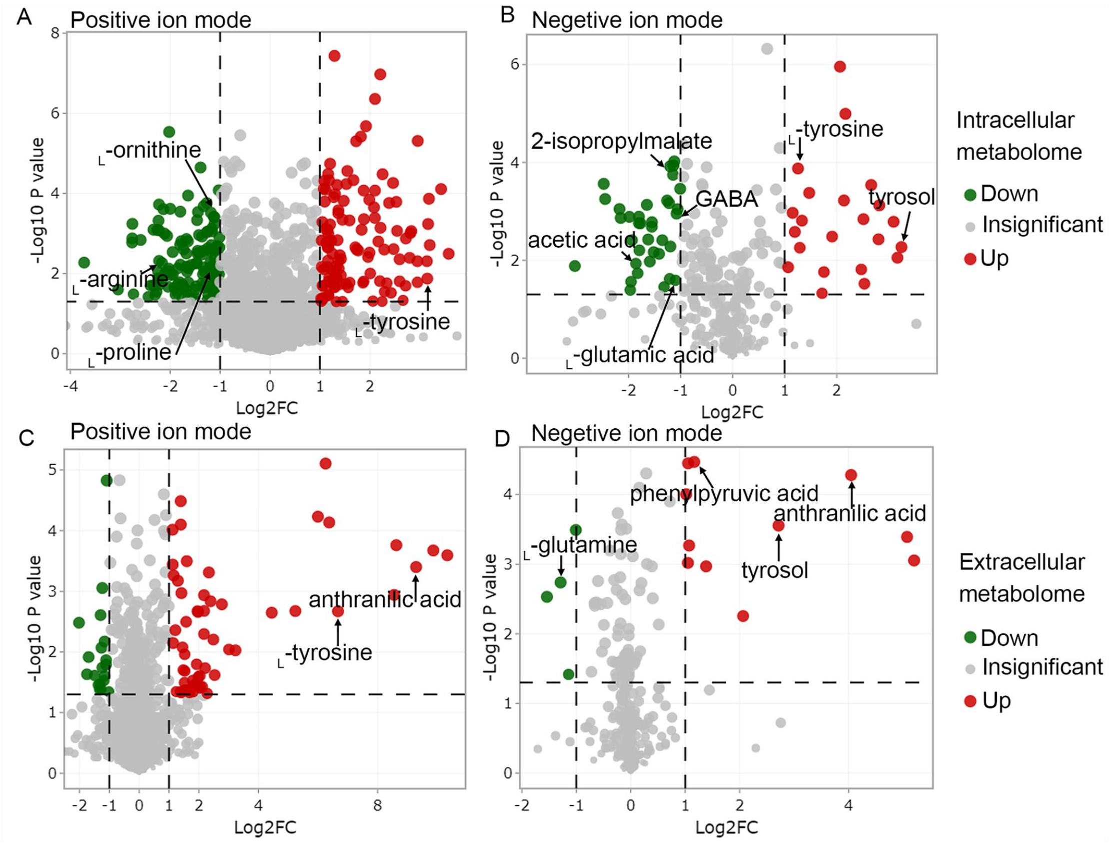

Supplementary Figure 9. Volcano plot based on the statistical significance of the OPLS-DA and fold change from the comparison of the metabolites between the wild type CEN.PK2-1C strain and the A3_3 strain. The identified metabolites of the aromatic amino acids biosynthetic pathway with significantly different concentrations in the A3_3 strains (overexpressing Aro3^{D154N}) in comparison to the WT strains were pointed out. The positive ion mode and the negative ion mode were used to detect the intracellular and extracellular metabolites between the two strains. The statistical analysis was carried out by R program. Orthogonal partial least square-discriminate analysis (OPLS-DA) was applied to distinguish the overall difference in the metabolic profile and to find differential metabolites between the groups. The comparisons of the intracellular metabolites based on the results of the positive ion mode and the negative ion mode were shown in (A) and (B), respectively. The comparisons of the extracellular metabolites based on the results of the positive ion mode and the negative ion mode were presented in (C) and (D), respectively. The downregulated metabolites were colored in green; the upregulated metabolites were shown in red and the metabolites with insignificant changes were shown in grey.

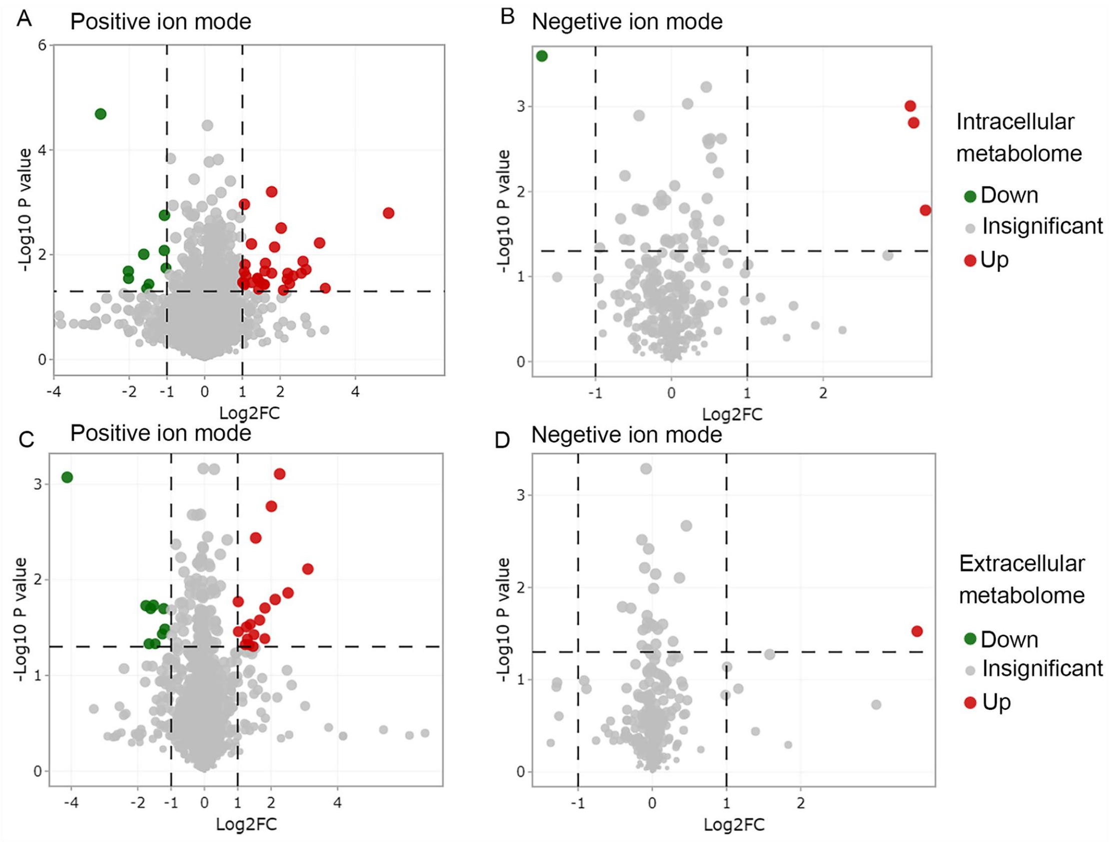

Supplementary Figure 10. Volcano plot based on the statistical significance of the OPLS-DA and fold change from the comparison of the metabolites between the WT CEN.PK2-1C strain and the A3_4 strain. The metabolites that pointed out in Supplementary Figure8 were insignificant difference metabolites between the WT CEN.PK2-1C strain and the A3_4 strain (overexpressing Aro3).

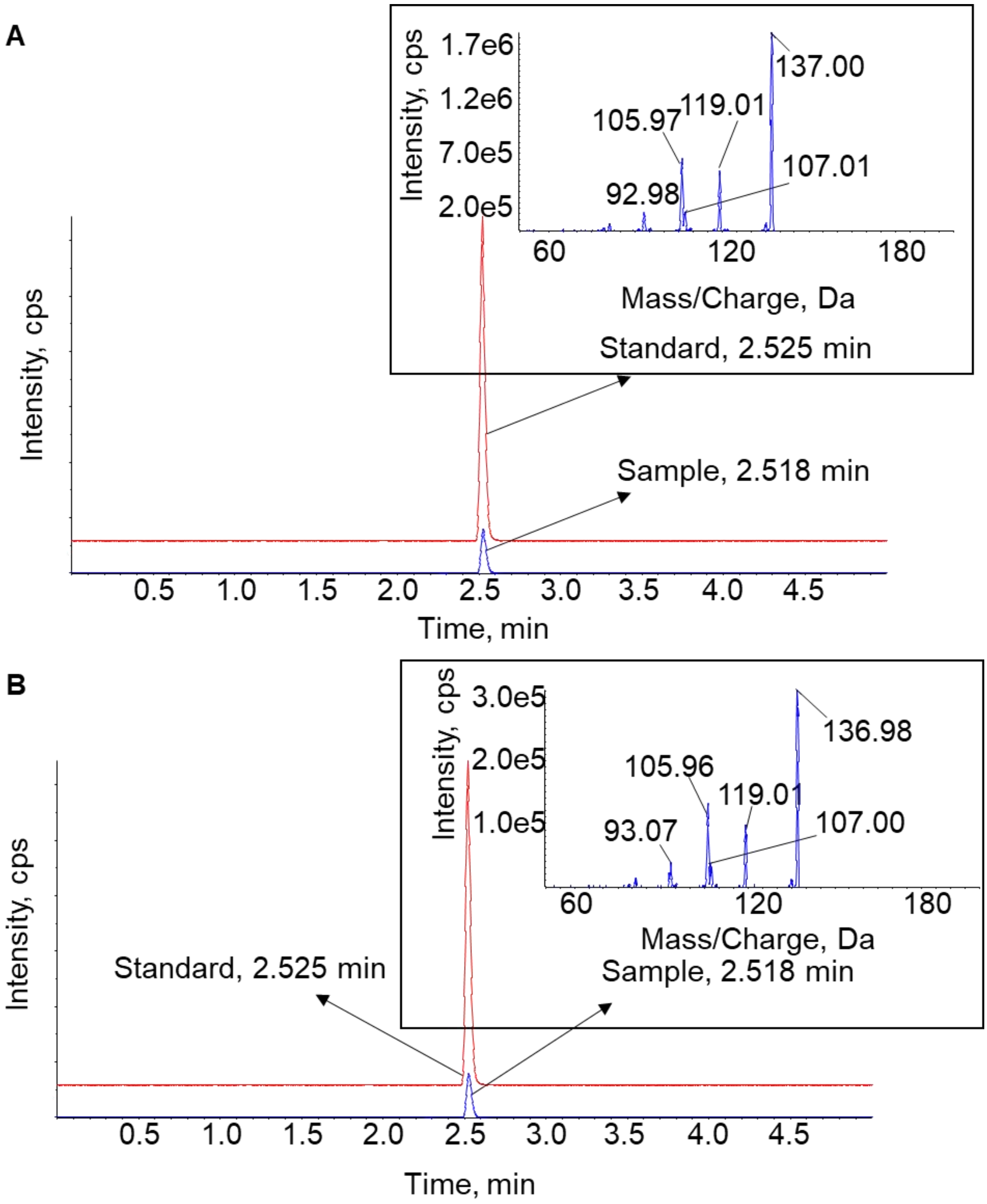

Supplementary Figure 11. The LC-MS analysis of standard tyrosol (A) and fermentation supernatant extracts (B).

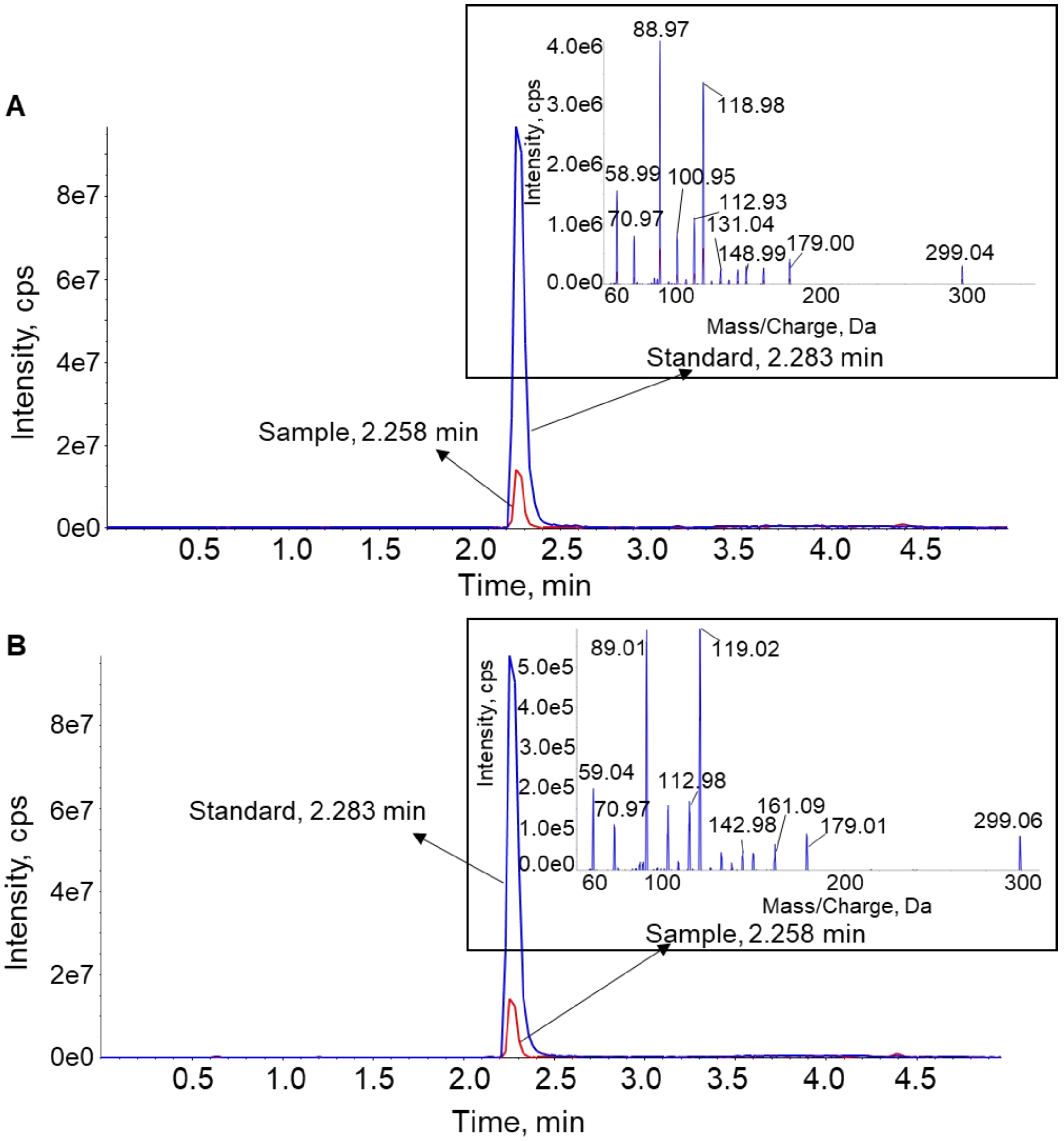

Supplementary Figure 12. The LC-MS analysis of standard salidroside (A) and fermentation supernatant extracts (B).

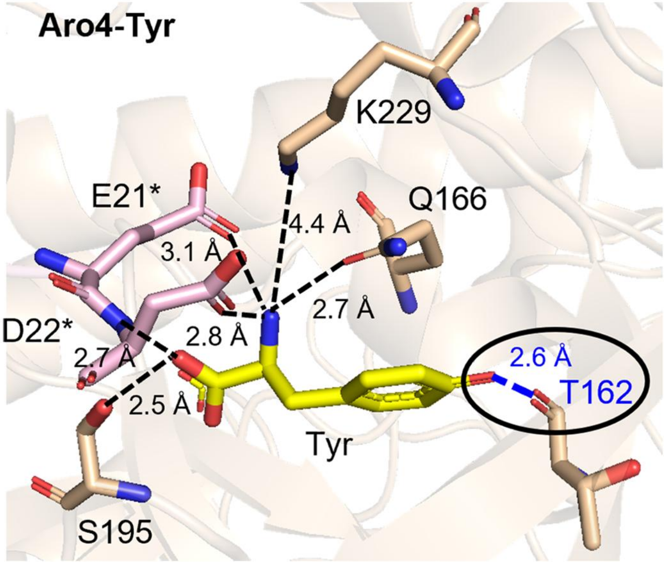

Supplementary Figure 13. The tyrosine-binding residues observed in the complex structure of Aro4-Tyr. The side chains of the Tyr binding residues from the complex structure of Aro4-Tyr (PDB: 1OF6) are shown as sticks. Tyr was shown as yellow sticks. The residues marked as asterisk (*) belong to the other subunits of the tight dimer. Black dotted lines indicate hydrogen bonds. Oxygen and nitrogen are shown in red and blue. The interaction of Tyr and T162 of Aro4 was highlighted as black circle.

Supplementary Table 1. *S. cerevisiae* strains used in this study.

Yeast Strains	Description
CEN.PK2-1C	*MAT***a** *ura3-52 his3-Δ1 leu2-3_112 trp1-289, MAL2-8c SUC2*
A3_1	CEN.PK2-1C P_{TEF1}-*ARO3* Δ*aro4*
A3_2	CEN.PK2-1C P_{TEF1}- *ARO3*^{K222L} Δ*aro4*
A3_3	CEN.PK2-1C P_{TEF1}- *ARO3*^{D154N} Δ*aro4*
A3_4	CEN.PK2-1C *TRP1* P_{TEF1}-*ARO3* Δ*aro4*
A3_5	CEN.PK2-1C *TRP1* P_{TEF1}- *ARO3*^{K222L} Δ*aro4*
A3_6	CEN.PK2-1C *TRP1* P_{TEF1}- *ARO3*^{D154N} Δ*aro4*
A3_7	CEN.PK2-1C *TRP1* P_{TEF1}-*ARO4* Δ*aro3*
A3_8	CEN.PK2-1C *TRP1* P_{TEF1}-*ARO4*^{D161N} Δ*aro3*
A3_9	CEN.PK2-1C *TRP1* P_{TEF1}-*AroF* Δ*aro4* Δ*aro3*
A3_10	CEN.PK2-1C *TRP1* P_{TEF1}-*AroF*^{D147N} Δ*aro4* Δ*aro3*
A3_11	CEN.PK2-1C *TRP1* P_{TEF1}-*YlARO4* Δ*aro4* Δ*aro3*
A3_12	CEN.PK2-1C *TRP1* P_{TEF1}-*YlARO4*^{D153N} Δ*aro4* Δ*aro3*
A3_13	CEN.PK2-1C *TRP1* P_{TEF1}-*YlARO3* Δ*aro4* Δ*aro3*
A3_14	CEN.PK2-1C *TRP1* P_{TEF1}-*YlARO3*^{D157N} Δ*aro4* Δ*aro3*
A3_15	CEN.PK2-1C *TRP1* P_{TEF1}-*KpARO4* Δ*aro4* Δ*aro3*
A3_16	CEN.PK2-1C *TRP1* P_{TEF1}-*KpARO4*^{D151N} Δ*aro4* Δ*aro3*
A3_17	CEN.PK2-1C *TRP1* P_{TEF1}-*KpARO3* Δ*aro4* Δ*aro3*
A3_18	CEN.PK2-1C *TRP1* P_{TEF1}-*KpARO3*^{D154N} Δ*aro4* Δ*aro3*
A3_19	CEN.PK2-1C *TRP1* P_{TEF1}-*KmARO4* Δ*aro4* Δ*aro3*
A3_20	CEN.PK2-1C *TRP1* P_{TEF1}-*KmARO4*^{D153N} Δ*aro4* Δ*aro3*
A3_21	CEN.PK2-1C *TRP1* P_{TEF1}-*KmARO3* Δ*aro4* Δ*aro3*
A3_22	CEN.PK2-1C *TRP1* P_{TEF1}-*KmARO3*^{D154N} Δ*aro4* Δ*aro3*
A3_23	CEN.PK2-1C *TRP1* P_{TEF1}-*OpARO4* Δ*aro4* Δ*aro3*
A3_24	CEN.PK2-1C *TRP1* P_{TEF1}-*OpARO4*^{D170N} Δ*aro4* Δ*aro3*
A3_25	CEN.PK2-1C *TRP1* P_{TEF1}-*OpARO3* Δ*aro4* Δ*aro3*
A3_26	CEN.PK2-1C *TRP1* P_{TEF1}-*OpARO3*^{D167N} Δ*aro4* Δ*aro3*
TY1	CEN.PK2-1C *308a* **::** P_{TDH3}-*ARO4*^{K229L}-T_{FBA1}-P_{TEF2}-*ARO7*^{G141S}-T_{ADH2}
TY2	TY1 Δ*pdc1* Δ*pha2*
TY3	TY2 *URA3* **::** P_{PGK1}-*ARO2*-T_{GPD}-P_{CCW12}-*ARO10*-T_{ADH1}
TY4	TY3 *TRP1* **::** P_{HXT7}-*TKL1*-T_{ADH1}-P_{TEF1}-*RKI1*-T_{PGK1}
TY5	TY4 *LEU2* :: P_{TEF1}-*ARO3*^{D154N}-T_{PGK1t}
TY6	TY4 *LEU2* :: P_{TEF1}-*ARO3*-T_{PGK1t}
TY7	TY4 *LEU2* :: P_{TEF1}-*ARO3*^{K222L}-T_{PGK1t}
SA1	TY4 *HIS3* :: P_{TEF1}-*RrU8GT33*-T_{PGK1t}
SA2	SA1 *LEU2* :: P_{TEF1}-*ARO3*^{D154N}-T_{PGK1t}

Supplementary Table 2. Plasmids used in this study.

Plasmids	Description
**The integrated plasmids**	
pTY1	PUC19, P_{TDH3}-*ARO4*^{K229L}-T_{FBA1}, P_{TEF2}-*ARO7*^{G141S}-T_{ADH2}
pTY2	Integrative plasmid, PRS404, *TRP1*, P_{HXT7}-*TKL1*-T_{ADH2}, P_{TEF1}-*RKI1*-T_{PGK1}
pTY3	Integrative plasmid, PRS406, *URA3*, P_{PGK1}-*ARO2*-T_{GPD}, P_{TEF1}-*ARO10*-T_{PGK1}
pTY4	Integrative plasmid, PRS405, *LUE2*, P_{TEF1}-*ARO3*-T_{PGK1t}
pTY5	Integrative plasmid, PRS405, *LUE2*, P_{TEF1}- *ARO3*^{K222L}-T_{PGK1t}
pTY6	Integrative plasmid, PRS405, *LUE2*, P_{TEF1}-*ARO3*^{D154N}-T_{PGK1t}
**The plasmids for protein expression**	
pET21-Aro3	pET21, Aro3-C-6×HIS
pET21-Aro3^{K222L}	pET21, Aro3^{K222L}-C-6×HIS
pET21-Aro3^{D154N}	pET21, Aro3^{D154N}-C-6×HIS
**The CRISPR/Cas9 plasmids**	
pCas-308a	pCas, 2μm, KanMX, gRNA-308a
pCas-1622b	pCas, 2μm, KanMX, gRNA-1622b
pCas-pha2	pCas, 2μm, KanMX, gRNA-*pha2*
pCas-pdc1	pCas, 2μm, KanMX, gRNA-*pdc1*
pCas-ARO3-promoter	pCas, 2μm, KanMX, gRNA-ARO3 promoter

Supplementary Table 3. Primers used in this study.

Primer name	Primer sequence 5'→ 3'
**Primers for construction of expression plasmids**	
pET21b-ARO3-for	TGTTAGCAGCCGGATCTCAGTGGTGGTGGTGGTGGTGTTTCTTCAAGGCTTTTCTTCTG
pET21b-ARO3-rev	TAATTTTGTTTAACTTTAAGAAGGAGATATACATATGTTCATTAAAAACGATCACG
**Primers for construction of gene-cassette plasmids, integration plasmids and donor plasmids**	
HXT7p-TKL1-for	ACAAAAAGTTTTTTTAATTTTAATCAAAAAATGACTCAATTCACTGACATTGATAAG
ADH2t-TKL1-rev	TATAAATCGTAAAGACATAAGAGATCCGCTTAGAAAGCTTTTTTCAAAGGAGAAATTAG
TEF1p-RKI1-for	TCATTAGAAAGAAAGCATAGCAATCTAATCTAAGATGGCTGCCGGTGTCCCAAAAATTG
PGK1t-RKI1-rev	TTGATCTATCGATTTCAATTCAATTCAATTCACTTTTCGGTAACTTCAACACTACCG
PRS404-HXT7p-55bp-for	ACTATAGGGCGAATTGGGTACCGGGCCCCCCCACTTCTCGTAGGAACAATTTCGG
ZWF1-left-HR-for	TGCGTTGTCGACGGTATCGATAAGCTTGATATTCAAGGTGTGGTGGCCCCTTTTCTAAG
BamHI-zwf1-left-rev	ACCGTACTGGCCCAAGGATCCGGGCCACGGCCTTTAGAACCTTAAC
BamHI-zwf-right-for	CGGATCCTTGGGCCAGTACGGTAAATCTGAGGAC
PRS404-zwf-right-53bp-rev	ACCGCGGTGGCGGCCGCTCTAGAACTAGTGGGAAAGCCAAAATGTCACTGACC
ARO4-K229L-for	CACCATTTCATGGGTGTTACTTTGCATGGTGTTGCTGCTATCAC
ARO4-K229L-rev	GTGATAGCAGCAACACCATGCAAAGTAACACCCATGAAATGGTG
ARO7-G141S-for	GAATAACTTCAGTTCTGTTGCCACTAGAGATATAGAATG
ARO7-G141S-rev	TCTAGTGGCAACAGAACTGAAGTTATTCTTATCATCACCATCTCTTTTCG
ARO3-K222L-for	ATCATTACTTCCTTTCTGTCACACTACCAGGTGTCACTGCTATCGTG
ARO3-K222L-rev	TGCCCACGATAGCAGTGACACCTGGTAGTGTGACAGAAAGGAAGTAATGATCATGTG
ARO3-D154N-for	ATTGCTGGTGAAATGCTGAACACCATTTCTCCGCAGTTTTTGAGTGA
ARO3-D154N-rev	TGCGGAGAAATGGTGTTCAGCATTTCACCAGCAATGGGTAATTTTTCA
TDH3p-ARO4-for	AGAACTTAGTTTCGAATAAACACACATAAACAAACAAAATGAGTGAATCTCCAATGTTC

FBA1t-ARO4-rev	ACTATATCAATTAATTTGAATTAACCTATTTCTTGTTAACTTCTCTTCTTTGTC
TEF2p-ARO7-for	AATATACGGTCAACGAACTATAATTAACTAAACATGGATTTCACAAAACCAGAAACTG
ADH2t-ARO7-rev	ACTATAAATCGTAAAGACATAAGAGATCCGCTTACTCTTCCAACCTTCTTAGCAAG
TEF1p-ARO3-for	CATAGCAATCTAATCTAAGTTTTAATTACAAAATGTTCATTAAAAACGATCACGCCGGT
PGK1t-ARO3-rev	GATCTATCGATTTCAATTCAATTCAATCTATTTTTTCAAGGCCTTTCTTCTG
PUC19-TDH3p-for	AGTCACGACGTTGTAAAACGACGGCCAGTTCATTATCAATACTGCCATTTC
TEF2p-FBA1t-rev	TACTATATGTAAGTATACGGCCCCAAAGATGAGCTAGGCTTTTGTAAAAATATC
FBA1t-TEF2p-for	AGATATTTTTACAAAAGCCTAGCTCATCTTTGGGGCCGTATACTTACATATAGTAGATG
PUC19-ADH2t-rev	AGCTTGCATGCCTGCAGGTCGACTCTAGAATGAGAAATATCGAGGGACTCGATTCAGAG
PUC19-TPI1t-rev	AGCTTGCATGCCTGCAGGTCGACTCTAGATATATAACAGTTGAAATTTGGATAAGAAC
PRS406-CCW12p-for	CGGTATCGATAAGCTTGATATCGAATTCGGATACTTCATGCTATTTATAGACGCGCGTG
CCW12p-ARO10-for	TCTTCTGTCATTCGCTTAAACACTATATCAATAATGGCACCTGTTACAATTGAAAAG
ADH1t-ARO10-rev	ATAAAAATCATAAATCATAAGAAATTCGCCTATTTTTTATTTCTTTTAAGTGCCGCTG
PGK1p-ADH1t-rev	GATGCGTTCATGCCTGACCGGTAGAGGTGTGGTCAATAAG
ADH1t-PGK1p-for	ACCACACCTCTACCGGTCAGGCATGAACGCATCACAG
TEF1p-GPDt-rev	AGCTATGGTGTGTGGGGAATCTGTGTATATTACTGCATCTAGATATATG
GPD1t-rev	GGAATCTGTGTATATTACTGCATCTAG
GPDt-ENO2p-for	CTAGATGCAGTAATATACACAGATTCCGTGTCGACGCTGCGGGTATAG
PRS406-TEF1t-rev	ACTAAAGGGAACAAAAGCTGGAGCTCCACCGCGGTGATAGCGCCGATCAAAGTATTTG
PGK1t-ENO2p-for	GTTTAATAACTCGAAAATTCTGCGTTACTAGTTCTAGAGTGTCGACGCTGCGGGTATAG
pUC19-ARO3-LHR-for	GGTTTTCCCAGTCACGACGTTGTAAAACGACGGCCAGTATCCTTGCGTTTCAGCTTCC
TEF-ARO3-LHR-rev	AAGGAGTAGAAACATTTTGAAGCTATGGTGTGTGGGTTGTTGTGGGGCATAATGCA
ARO3-LHR-TEF1p-for	TCATCAAAATATGCATTATGCCCCACAACAACCCA

	CACACCATAGCTTCAAAATGT
	ATTTGCATTTTTTCTTGTTTGAAGTTGAATTCTTTG
ARO3-RHR-TEF-rev	TAATTAAAACTTAGATTAGATTGCTATGC
	CTAATCTAAGTTTTAATTACAAAGAATTCAACTTCA
TEF-ARO3-RHR-for	AACAAGAAAAAATGCAAATAATCTTG
	AAGCTTGCATGCCTGCAGGTCGACTCTAGAGTAT
pUC19-ARO3-RHR-rev	AAACTTCTCGATAAACAAAAGGTTCCA
**Primers for construction of donor DNA**	
	TATTTCAGAAAAATTATTCAAAACTAAGAAGAATGA
308a-left-HR-for	GATG
308a-left-HR-rev	TTAGATAAAAAGAAAAAAATTCGAAGTTAATGTTG
	CAACATTAACTTCGAATTTTTTTCTTTTTATCTAATC
308a-TDH3p-ARO4-for	ATTATCAATACTGCCATTTC
308a-right-HR-rev	TGATAGAACGAGTACAACACCCGA
308a-right-HR-for	TTCTTTGCTACATATTGCTACCACTTCTATTAC
1622b-left-for	AACATTTAAGTCACAAGGAGGAATATCAGTT
1622b-left-rev	AACTACTTTTCTTAAACTGTCAACAGCCA
1622b-left-PDC1p-for	TCATTGGCTGTTGACAGTTTAAGAAAAGTAGTTCA
	TGCGACTGGGTGAGCATATGTT
1622b-right-for	GTAGATACTCGTCTTACGAAATTGGATATAGTT
1622b-right-PGK1t-rev	AACTATATCCAATTTCGTAAGACGAGTATCTACCA
	GGAAGAATACACTATACTGGATCT
1622b-right-rev	ACTTTGGAAAAGAAGGTACGGACTACT
TEF1p-ARO3-for	CATAGCAATCTAATCTAAGTTTTAATTACAAAATGT
	TCATTAAAAACGATCACGCCGGT
PGK1t-ARO3-rev	GATCTATCGATTTCAATTCAATTCAATCTATTTTTTC
	AAGGCCTTTCTTCTG
L-ARO3pr-HR-for	AATCCCTACATTCAGATTCC
TEF1p-LARO3pr-rev	AGAAACATTTTGAAGCTATGGTGTGTGGATGACTT
	TCGAAGATTGTATTTTTC
LARO3pr-TEF1p-for	AGTCATCCACACACCATAGCTTCAAAATG
ARO3-TEF1p-rev	TGATCGTTTTTAATGAACATTTTGTAATTAAAACTT
	AGATTAGATTGCTATGCT
TEF1p-RARO3-for	AATTACAAAATGTTCATTAAAAACGATCAC
RARO3HR-rev	TCTCTCGAAATCCGTAGACC
TEF-aroH-for	CATAGCAATCTAATCTAAGTTTTAATTACAAAATGA
	ACAGAACTGACGAACTCC
aroH-2-D145N-LRH-rev	GATTAAATCAGCAATAAACTGACCGGTCACCATAT
	TGAGGAACTCGGTCGCGGTTG
aroH-2-D145N-RHR-for	GGCGTCCCAACCGCGACCGAGTTCCTCAATATG
	GTGACCGGTCAGTTTATTG
ARO3HR-aroH-rev	ATTATTTGCATTTTTTCTTGTTTGAAGTTTCAGAAG
	CGGGTATCTACCG
TEF-aroF-for	CATAGCAATCTAATCTAAGTTTTAATTACAAAATGC

	AAAAAGACGCGCTGAATA
aroF-2-D147N-LRH-rev	ACAGATCGCCCAGGTATTGCGGGCTATTCGGATTTAACGCTTCCGTCGCCAGTG
aroF-2-D147N-RHR-for	GACTGCCACTGGCGACGGAAGCGTTAAATCCGAATAGCCCGCAATAC
ARO3HR-aroF-rev	TCAAGATTATTTGCATTTTTTCTTGTTTGAAGTTTTAAGCCACGCGAGCCGTCA
TEF-*YlARO4*-for	ATAGCAATCTAATCTAAGTTTTAATTACAAAATGTCCCGTTCCTCCTCTCC
YlARO4-2-D153N-LRH-rev	CCATTGCCTCCGAGATGCTCAACACCATTTCTCCCCAGTTCCTGGCCGACCTGCT
YlARO4-2-D153N-RHR-for	TTGGGTCTGCCCATTGCCTCCGAGATGCTCAACACCATTTCTCCCCAGTTCC
ARO3HR-*Yl*ARO4-rev	TCAAGATTATTTGCATTTTTTCTTGTTTGAAGTTTTAGTTCTTGTTTCGTCGCTCCT
TEF-*Yl*ARO3-for	GCATAGCAATCTAATCTAAGTTTTAATTACAAAATGCCCGCTATGCACAACGC
*Yl*ARO3-2-D157N-LRH-rev	GAGAAGAGATCAGCCAGGAACTGGGGAGAAATGGTGTTCAGAAGCTCAGAAGCAATGGG
*Yl*ARO3-2-D157N-RHR-for	AGCTCAACCCCATTGCTTCTGAGCTTCTGAACACCATTTCTCCCCAGTTCC
ARO3HR-*Yl*ARO3-rev	ATTCAAGATTATTTGCATTTTTTCTTGTTTGAAGTTCTAACCTCGTCGAGTCTTGACG
TEF-*Kp*ARO4-for	AAAGCATAGCAATCTAATCTAAGTTTTAATTACAAAATGACCTCCACACCAGTTCA
*Kp*ARO4-2-D151N-LRH-rev	TAGCAAATCAGCCAAGTATTGAGGGGAAATTGTGTTCAACATTTCACTGGCAATAGGC
*Kp*ARO4-2-D151N-RHR-for	AACAGCGGTATGCCTATTGCCAGTGAAATGTTGAACACAATTTCCCCTCAATACTTG
ARO3HR-*Kp*ARO4-rev	TTCAAGATTATTTGCATTTTTTCTTGTTTGAAGTTCTAAGCAGCGTTCTTTAATGCT
TEF-*Kp*ARO3-for	AAAGCATAGCAATCTAATCTAAGTTTTAATTACAAAATGTTCATTCAAAACGATCATGTC
*Kp*ARO3-2-D154N-LRH-rev	GAGAAGAGATCAGACAGGAATTGAGGGGAGATAGTGTTCAGCATCTCACCAGCAATAG
*Kp*ARO3-2-D154N-RHR-for	AACAGATTCCTATTGCTGGTGAGATGCTGAACACTATCTCCCCTCAATTCC
ARO3HR-*Kp*ARO3-rev	TCAAGATTATTTGCATTTTTTCTTGTTTGAAGTTCTAATTCTTGAGATTACGACGTTCA
TEF-*Km*ARO4-for	GAAAGCATAGCAATCTAATCTAAGTTTTAATTACAAAATGTCTGCTACTCCACAACC
*Km*ARO4-2-D153N-LRH-rev	ACAAATCAGCCAAAAATTGTGGAGAAATAGTATTCAACATTTCAGAACCAATTGGC
*Km*ARO4-2-D153N-RHR-for	GTTTGCCAATTGGTTCTGAAATGTTGAATACTATTT

	CTCCACAATTTTTGGC
ARO3HR-*Km*ARO4-rev	TCAAGATTATTTGCATTTTTTCTTGTTTGAAGTTTTATTTAGCAGCTTTCTTTTTCAATTC
TEF-*Km*ARO3-for	AAAGCATAGCAATCTAATCTAAGTTTTAATTACAAAATGTTTATTTCTAATGATAGAATTGGTG
*Km*ARO3-2-D154N-LRH-rev	AACAATCAGACAAAAATTGTGGAGAAATAGTATTCAACATTTCACCAGCAATTG
*Km*ARO3-2-D154N-RHR-for	AAATTGCCAATTGCTGGTGAAATGTTGAATACTATTTCTCCACAATTTTTGTC
ARO3HR-*Km*ARO3-rev	AGATTATTTGCATTTTTTCTTGTTTGAAGTTTTATTTTTTCAAAGCAGCTCTTC
TEF-*Op*ARO4-for	AAGAAAGCATAGCAATCTAATCTAAGTTTTAATTACAAAATGTCTGCTGTTGCTGGTTC
*Op*ARO4-2-D170N-LRH-rev	ATCAGCCAAAAATTGTGGAGAAATAGTATTCAACATTTCAGAACCAATTGGCATAC
*Op*ARO4-2-D170N-RHR-for	AATGAAGGTATGCCAATTGGTTCTGAAATGTTGAATACTATTTCTCCACAATTTTTGGC
ARO3HR-*Op*ARO4-rev	TCAAGATTATTTGCATTTTTTCTTGTTTGAAGTTTTATTTCAAAGTTCTTCTAATTTTAACAGCT
TEF-*Op*ARO3-for	AAGAAAGCATAGCAATCTAATCTAAGTTTTAATTACAAAATGTTGACTAATCAAGCTCCAAC
*Op*ARO3-2-D167N-LRH-rev	AACAAATCAGACAAAAATTGTGGAGAAATAGTATTCAACATTTCACCAGCAATTGG
*Op*ARO3-2-D167N-RHR-for	TGAAAAATTGCCAATTGCTGGTGAAATGTTGAATACTATTTCTCCACAATTTTTGTC
ARO3HR-*Op*ARO3-rev	CATTCAAGATTATTTGCATTTTTTCTTGTTTGAAGTTTTAATTTTTATTTTTCAAAGATCTTCTTTCT
**Primers for construction of CRISPR/Cas9 plasmids**	
pCas-308a-for	CTTTGGTCTCACTTTCACTTGTCAAACAGAATATAGTTTAGAGACCTTTC
pCas-308a-rev	GAAAGGTCTCTAAACTATATTCTGTTTGACAAGTGAAAGTGAGACCAAAG
pCas-1622b-for	CTTTGGTCTCACTTTCCTGAGGTTACTGGGGACAAGTTTAGAGACCTTTC
pCas-1622b-rev	GAAAGGTCTCTAAACTTGTCCCCAGTAACCTCAGGAAAGTGAGACCAAAG
pCas-pdc1-for	CTTTGGTCTCACTTTAGTCACCGTTACCCAAGGTGGTTTAGAGACCTTTC
pCas-pdc1-rev	GAAAGGTCTCTAAACCACCTTGGGTAACGGTGACTAAAGTGAGACCAAAG
pdc1-donor-for	TGTTTTGCACGTTGTTGGTGTCCCATCCATCTCTTCTCAAGCTAAGCAATTGTTGTTG
pdc1-donor-rev-1	AGAAATGTTGGCAGACATTCTGTGGAAAACAGTGATGCAACAACAATTGCTTAGCTTGA

pdc1-donor-rev-2	CAATGTCAGTGATCATAGCAGTGGTTTCAGAAATGTTGGCAGACATTCTG
pCas-pha2-for	CTTTGGTCTCACTTTGGGGGATAGAGGCTGCTGGGGTTTAGAGACCTTTC
pCas-pha2-rev	GAAAGGTCTCTAAACCCCAGCAGCCTCTATCCCCCAAAGTGAGACCAAAG
pha2-donor-for	AGGTACGTATTCCCATCAAGCTGCATTACAACAATTTCAATCAACATCTGATGTTGAG
pha2-donor-rev-1	CCAACGGTACCACTGAATAATCTATACTAGTGTCGTTCTCCAATTGGTTAAAACATTTA
pha2-donor-rev-2	CAATTTCAATCAACATCTGATGTTGAGTAAATGTTTTAACCAATTGGAGAACGACAC
pCas-ARO3N-for	CTTTGGTCTCACTTTACCCATTGCTGGTGAGATGTGTTTAGAGACCTTTC
pCas-ARO3N-rev	GAAAGGTCTCTAAACACATCTCACCAGCAATGGGTAAAGTGAGACCAAAG
pCas-ARO3L-for	CTTTGGTCTCACTTTTTCCTTTCTGTCACAAAGCCGTTTAGAGACCTTTC
pCas-ARO3L-rev	GAAAGGTCTCTAAACGGCTTTGTGACAGAAAGGAAAAAGTGAGACCAAAG
pCas-ARO3-promoter-for	CTTTGGTCTCACTTTACAGGCAAGTGATTTTACGGGTTTAGAGACCTTTC
pCas-ARO3-promoter-rev	GAAAGGTCTCTAAACCCGTAAAATCACTTGCCTGTAAAGTGAGACCAAAG
**Primers for sequencing**	
seq-pCas-lacZ-for	TCGGAATAGGAACTTCAAAGCG
seq-1622b-for	AGCTCTTGTGGCACCTATAG
seq-1622b-rev	TACATGGATTGAGCATTAGAGAG
seq-pdc1-for	ACGAATTGAACGCTGCTTAC
seq-pdc1-rev	TTCAGCCTTGACGTCGTGTC
seq-pha2-for	AACGAAGAATTACTTCCGTATACG
seq-pha2-rev	TTATTTGTGATAATATCTCTCATTTCTGGG
seq-ARO3N-out-for	AAGACTGGAGAATCAAAGGT
seq-ARO3N-rev	TACCACCTCTCAAGATCAGG
seq-ARO3-TEF1-out-for	TCTGCACAAGGGAATTGACA
seq-ARO3-TEF1-out-rev	TCTGTGCAGTTGGGATTCAG
seq-ARO3HR-out-for	ACGATTACCGTATTATCCACC
seq-ARO3HR-out-rev	AGATTGCCTTGAGATTGGTG
seq-ARO3-LHR-in-for	GCAATACCAAAATGAGGCATC
seq-aroH-in-3-for	ACAGAACTGACGAACTCC
seq-aroF-in-3-for	GTACATATTACCGACGAACAGG
seq-*Yl*ARO4-in-3-for	CGAAAGGACTCCATTGATGTC
seq-*Yl*ARO3-in-3-for	GAATCCGGGGCTACAACC
seq-*Kp*ARO4-in-3-for	TACGACGATGTACGTATAGCG

seq-*Kp*ARO3-in-3-for	ACAGATCCCGCTTAGAAGAC
seq-*Km*ARO4-in-3-for	GTTGCAAGCTCAAGTTCCTG
seq-*Km*ARO3-in-3-for	GTTATGATCCATTGACTCCACC
seq-*Op*ARO4-in-3-for	AGCTAGATCTTCTACTCCTGC
seq-*Op*ARO3-in-3-for	TGACTAATCAAGCTCCAACTG
