# Supplementary material for: Mechanistic investigation of a D to N mutation in DAHP synthase that dictates carbon flux into the shikimate pathway in yeast
Source: Commun Chem. 2023 Jul 15;6:152. doi: 10.1038/s42004-023-00946-x (PMC10349828; doi:10.1038/s42004-023-00946-x)
Supplement: Supplementary file 2 — Description of Additional Supplementary Files [file 42004_2023_946_MOESM2_ESM.pdf]

Description of Additional Supplementary Files

File name: Supplementary Data 1

Description: Protein crystal data

File name: Supplementary Data 2

Description: The 500 structural coordinates of the initial conformation and every 100 ns conformation 501 obtained

File name: Supplementary Data 3

Description: Gene 502 sequences

File name: Supplementary Data 4

Description: Source data
