# Supplementary material for: Mechanistic investigation of a D to N mutation in DAHP synthase that dictates carbon flux into the shikimate pathway in yeast
Source: Commun Chem. 2023 Jul 15;6:152. doi: 10.1038/s42004-023-00946-x (PMC10349828; doi:10.1038/s42004-023-00946-x)
Supplement: Supplementary file 5 — Supplementary Data 3 [file 42004_2023_946_MOESM5_ESM.docx]

**DNA sequences of Type Iα DAHPS used in this study**

>*KmARO4* from *Kluyveromyces marxianus* strain DMKU3-1042

ATGTCTGCTACTCCACAACCAATGTTTCATGAACAAGAAGATGTTAGAATTTTGGGTTATGATCCATTGGTTTCTCCTGCTTTGTTGCAAGCTCAAGTTCCTGCTTCTCCTGAATGTTTGGCTACTGCTCAAAGAGGTAGAAAAGAATCTGTTGATATTATTACTGGTAAAGATGATAGAGTTTTGGTTATTGTTGGTCCATGTTCTATTCATGATTTGGATCAAGCTCAAGAATATGCTAAGATGTTGAAGGCTTTATCAGACGAATTAAAAGATGATTTGTGCATCATTATGAGAGCTTATTTGGAAAAACCAAGAACTACTGTTGGTTGGAAAGGTTTAATCAATGACCCTGATGTCGACAATACATTCAATATCAATAAAGGTTTGCAAGTTTCTAGGCAATTGTTTGTTAATTTGACTTCTTTGGGTTTGCCAATTGGTTCTGAAATGTTGGATACTATTTCTCCACAATTTTTGGCTGATTTGTTGTCTTTTGGTGCTATTGGTGCTAGAACTACTGAATCTCAATTGCATAGAGAATTGGCTTCTGGTTTGTCTTTTCCTGTTGGTTTTAAAAATGGTACTGATGGTACTTTGGGTGTTGCTGTTGATGCTGTTCAAGCTGCTTCTCATCCACATCATTTTATGGGTGTTACTAAACATGGTGTTGCTGCTATTACTACAACTAAAGGTAATGAACATTGTTTTGTTATTTTGAGAGGTGGTAAAAAAGGTACTAATTATGATCCTGCTTCTGTTGCTGAAGCTAAAGCTCAATTGCCTGAAAAGGGTGTTTTGATGATCGACTACTCTCATGGTAACTCTAATAAAGATTTTAGAAATCAACCAAAAGTTAATGATGTTGTTTGTGAGCAGATTGCTAATGGTGAGGATAAGATTATTGGTGTTATGATTGAATCAAACATTAATGAAGGTAAACAATGTATTCCACCTGAAGGTAAAGCTGGTTTGAAATATGGTGTTTCAATTACTGATGGTTGTATTTCTTTCGAAACTACAACTGAAGTTTTGAGAAAATTGGCTGCAGCTGTTAGAGCTAGAAGAGAATTGAAAAAGAAAGCTGCTAAATAA

>*KmARO3* from *Kluyveromyces marxianus* strain DMKU3-1042

ATGTTTATTTCTAATGATAGAATTGGTGATAGATCTTCTTTGGAAGATTGGAGAATTAAAGGTTATGATCCATTGACTCCACCTGATTTGTTGCAGCACGAATACCCAATTGGAGAAAAAGCTAAAAAAATTATTGTCGATGCTAGAAATCAAGTCTGTGATATCTTGAATGGTAAAGATGATAGATTGGTTGTCGTTATTGGTCCATGTTCTATTCATGATCCAAAAGCTGCTTATGAATATGCTGATAGATTGAAAAAATTGTCTGATGAATTGTCTGGTGATTTGTTGATTATTATGAGAGCTTATTTGGAAAAACCAAGAACTACTGTCGGTTGGAAAGGATTGATCAATGACCCTGACATTGACAATTCTTTTCAAATTAATAAAGGTTTAAGAATTTCTAGAGAAATGTTTACTAGATTGACTGAAAAATTGCCAATTGCTGGTGAAATGTTGGATACTATTTCTCCACAATTTTTGTCTGATTGTTTTTCTTTGGGTGCTATTGGTGCTAGAACTACTGAATCTCAATTGCATAGAGAATTGGCTTCTGGTTTGTCTTTTCCAATTGGTTTTAAAAATGGTACTGATGGTGGTTTGCAAGTTGCTGTTGATGCTATGAGAGCTGCAGCTCATTCTCATTATTTTTTGTCTGTTACTAAACCTGGTGTTACTGCTATTGTTGGTACTGAAGGTAATGAAGATACTTTTGTTATTTTGAGAGGTGGTAAAAAAGGTACTAATTATGATGCTGAAAATGTTAAATTGGCTCAACAAGAATTGTTGAAAGCTAAAGTTGTTGATGCTGAAGGTGTTCAAAGAAGAATTATGATTGATTGTTCTCATGGTAATTCTTCTAAAGATTTTAGAAATCAGCCAAAAGTTGCTCAATCTATTTATGAACAATTGACTGATGGTCAAAATGCTATTTGTGGTGTTATGATTGAATCTAATTTGGTTGAAGGTAGACAAGATATTCCACCTGAAGGTGGTAGAGATAAATTGATTTATGGTTGTTCTATTACTGATGCTTGTATTGGTTGGGATACTACTGATGAAGTTTTGAGATTGTTGGCTAAGGGAGTTCAAAATAGAAGAGCTGCTTTGAAAAAATAA

>*OpARO4* from *Ogataea parapolymorpha* strain ATCC 26012 (*Hansenula polymorpha*)

ATGTCTGCTGTTGCTGGTTCTCATAAAAATAATGAATTGTTGGAATTTGAAAGAGCTAGATCTTCTACTCCTGCTCCTGAAGAATATGATGATGTTAGAATTGCTGGTTATGAACCATTGGTTTCTCCTGCTTTGTTGCAACAAGAAGTTCCACCAACTAAGGCTTCTTTGGCTGCTGTTATTAAAGGTAGAAAAGCTGCTTCTGCTGTTGTTAAACAACAAGATGATAGATTGTTGGTTGTCGTTGGTCCATGTTCTTTGCATGATCCTGTTGCTGCTATGGATTATTGTAGAAGATTGAAAAAATTGTCTGATGAATTGTCTGGTGAATTGGTTGTTATTATGAGAGCTTATTTGGAAAAACCAAGAACTACTGTTGGTTGGAAGGGTTTGATCAATGACCCTGATTTGGATACAACTTTTAATATCAATAAAGGTTTGAAAATTTCTAGAAAATTGTTTGTTGAATTGACTAATGAAGGTATGCCAATTGGTTCTGAAATGTTGGATACTATTTCTCCACAATTTTTGGCTGATTTGTTGTCTTTTGGTGCTATTGGTGCTAGAACTACTGAATCTCAATTGCATAGAGAATTGGCTTCTGGTTTGTCTTTTCCAATTGGTTTTAAAAATGGTACTGATGGTTCTTTGGATGTTGCTATTGATGCTATTCAAGCTGCAGCTTCTCCACATCATTTTATGGGTGTTACTAAACATGGTTTAGCTGCTATTACTACAACTAAAGGTAATGATAATTGCTTCATTATTTTGAGAGGTGGTAAAAAAGGTACTAATTATGATGAGGCTTCTGTTGCTGAAGCTAAATCTAAATTGCCACCTGGTGGAGTTTTGATGGTTGATTGTTCTCACGGTAATTCTAATAAAGACTATAGAAATCAGCCAAAAGTTTCTAAAGAAGTTGCTAGACAAATTGCTAAAGGTGAAGACAAAATTATTGGTGTTATGATCGAATCTCATATTAATGAGGGAAAACAGTCTATTCCAAAAGATAAATCTGAATTGAAATATGGTGTTTCTGTTACTGATGGTTGTGTTTCTTGGGAAACTACTGTTGAAATGTTGACTGAATTGGCTGAAGCTGTTAAAATTAGAAGAACTTTGAAATAA

>*OpARO3* from *Ogataea parapolymorpha* strain ATCC 26012 (*Hansenula polymorpha*)

ATGTTGACTAATCAAGCTCCAACTGCTTCTCATTCTAGAATGTTTATGGAAAATCCACATGTTGGTGATAGATCTAGATTGGAAGATTGGAGAATTAGAGGTTATAATCCATTGACTCCACCTGATTTATTGCAACATGAATATCCATTGACTTCTAAATCTGAGAAGAATATTTTGGAAGGTAGAGAAGATGCTTGTAATATTTTGAATGGTAAAGATGATAGATTGTTGGTTGTTATTGGTCCATGTTCTTTGCATGATCCTGAAGCAGCATTGGACTATTGCAATAGATTATCTGCATTCAAAGAGAAGGTTAAAGGTGAATTGCATATTGTTATGAGAGCTTATTTGGAAAAACCAAGAACTACTGTTGGTTGGAAAGGTTTAATTAATGACCCTGATATTGACGGTTCATTCAATATTAACAAAGGATTGAGAATTGCTAGAGAATTGTTTGTTAGATTGACTGAAAAATTGCCAATTGCTGGTGAAATGTTGGATACTATTTCTCCACAATTTTTGTCTGATTTGTTTTCTGTTGGTGCTATTGGTGCTAGAACTACTGAATCTCAATTGCATAGAGAATTGGCTTCTGGTTTGTCTTTTCCTGTTGGTTTTAAAAATGGTACTGATGGTTCTTTGGGTGTTGCTGTTGATGCTATGAGAGCTGCAGCTCATCCACATCATTTTTTGTCTGTTACTAAACCTGGTGTTGTTGCTATTGTTGGTACTGAGGGTAATAAAGATACTTTTATTATTTTGAGAGGTGGTAAAAAAGGTACTAATTATGATGAACAATCTGTTAAAGAAGCTGAACAAGCATTGTCTAAAGCTGGTATTTTGAAAGAAGGTGAAGCTAGAATTATGGTTGATTGTTCTCATGGTAATTCTTGTAAAGATCATAGAAATCAACCAAAAGTCGCAGCAGAAATCTCAAGACAATTAAAAAATGGTAATACTACAATTTGCGGAGTTATGATTGAGTCTAATATTAATGAAGGTAGACAAGATGTTCCACCATTGGAAGAAGGTGGTAAAGATTGTTTGAAATATGGTTGTTCTATTACTGATGCTTGTATTGGTTGGGAATCTACTGAAGAAGTTTTGGAAATGTTGGCTGAAGCTGTTAAAGAAAGAAGATCTTTGAAAAATAAAAATTAA

>*YlARO4* from *Yarrowia lipolytica*

ATGTCCCGTTCCTCCTCTCCCAACGCCTCCTCTGCTGAGGACGTGCGAATTCTGGGCTACGACCCCCTCCTCGCTCCCGCTCTTCTCCAGACTGAGGTTGCCTCCACCAAAAACGCCCGAGAGACCGTCTCCAAGGGCCGAAAGGACTCCATTGATGTCATCACCGGCAAGTCCGACAAGTTGCTGTGCATTGTCGGTCCCTGCTCCCTCCACGACCCCAAGGCCGCCATGGAGTACGCCCAGCGACTCAAGGAGCTGTCTGACAAGCTGTCTGGTGAGCTCGTCATCGTTATGCGAGCCTACCTCGAGAAGCCCCGAACCACCGTTGGCTGGAAGGGTCTGATCAACGACCCCGACATGGACGAGTCTTTCAACATCAACAAGGGTCTGCGACTCTCCCGAAAGGTCTTCTGCGACCTTACCGACTTGGGTCTGCCCATTGCCTCCGAGATGCTCGATACCATTTCTCCCCAGTTCCTGGCCGACCTGCTCTCCCTGGGTGCCATTGGTGCTCGAACCACCGAGTCCCAGCTGCACCGAGAGCTCGCCTCCGGTCTGTCTTTCCCCGTTGGTTTCAAGAACGGAACCGACGGTACTCTGGGTGTTGCCGTTGATGCTGTCCAGGCCGCCTCTCACCCTCACCACTTCATGGGTGTCACCAAGCAGGGTGTTGCCGCCATCACCACCACCAAGGGTAACGAGAACTGCTTCATCATTCTGCGAGGAGGTAAGAAGGGCACCAACTACGACGCCGAGTCCGTCGCCGAGTGCAAGAAGGCCACCGAGTCCATGCTCATGGTTGACTGCTCTCACGGCAACTCCAACAAGGACTACCGAAACCAGCCCAAGGTTTCCAAGGCCGTTGCTGAGCAGGTTGCTGCTGGCGAGAAGAAGATCATCGGTGTCATGATCGAGAGTAATATCCACGAGGGCAACCAGAAGGTCCCCAAGGAGGGCCCCTCTGCCCTTAAATACGGTGTCTCCATCACCGACGCCTGTGTCTCTTGGGAGACCACCGTGGACATGCTCACCGAGCTGGCCAACGCCGTCAAGGAGCGACGAAACAAGAACTAA

>*YlARO3* from *Yarrowia lipolytica*

ATGCCCGCTATGCACAACGCTTCTAACGCTCAGGGAGACCGAAACCGGACCGAGGACTGGCGAATCCGGGGCTACAACCCTCTCACAGCCCCCGATCTGCTCCAGCATGAGATCCCTCTGACCAAGCAGTCCAAGGCCACCATTCTCAAGGGCCGACAGGACGCCTGTGATATTCTGGATGGTAAGGACGACCGAATCATTGTTGTGGTTGGCCCCTGTTCCATCCATGACCCCAAGGCTGCCATGGAGTACGCCGAGCGACTCAAGCAGATCTCTGACAAGCTGTCTGGCGAGCTTCTGATCGTCATGCGAGCCTACCTCGAAAAGCCTCGAACCACCGTTGGCTGGAAGGGCCTTATTAATGATCCTGACATGGACGGCTCTTTCAAGATCAACAAGGGCCTGCGAGTCGCTAGAGATCTCTTCGTGAAGCTCACCGAGCTCAACCCCATTGCTTCTGAGCTTCTGGACACCATTTCTCCCCAGTTCCTGGCTGATCTCTTCTCTGTCGGAGCCATTGGTGCTCGAACCACCGAGTCCCAGCTTCACCGAGAGCTTGCCTCTGGTCTGTCTTTCCCCGTTGGTTTCAAGAACGGTACTGACGGAGGTATTAAGGTGGCTCTGGACGCCATCCAGGCCGCCGCCCACCCCCACCACTTCCTTTCCGTCACCAAGCCCGGTGTGGTTGCCATTGTCGGCACCGACGGAAACGAGGACTGCTTCCTGATTCTGCGAGGAGGTTCCAAGGGCCCCAACTACGATGCTGAGCACGTCGCCGAGGTCAAGAAGCAGGTTGGAGAGACCAAGGGTCCCCGAATCATGGTAGACTGCTCGCACGGCAACTCATCAAAGAACCACAAGAACCAGCCTCTGGTCGCCTCTAACGTGGCTCAGCAGATTGCTGCTGGTGAGAAGTCCATTTGCGGTCTTATGATTGAGTCCAACATCCACGAGGGTCGACAGGACATCTGCGATAACAAGGAGGACATGAAGTACGGTGTGTCTGTCACCGATGCCTGCATCAACTGGGAGGACACTGAGAAGGTGCTCGAGGAGCTGGCCCAGGCCGTCAAGACTCGACGAGGTTAG

>*KpARO4* from *Komagataella phaffii* GS115 (*Pichia pastoris*)

ATGACCTCCACACCAGTTCAAGAAGAATACGACGATGTACGTATAGCGGGATATGACCCATTGGTTTCCCCTGCTCTGCTTCAACAGGAGATTCCTGCCACCAAAGTTGCTCTTCAAACCGTGGTTAAAGGGAGAAAAGATGCCATGAACGTAGTTTCCCTGAAAGATGACAGATTATTGGTAGTTGTCGGACCATGTTCCATCCATGACAGTGACGCCGCTCTCGAGTACGTTGCCAGGTTGAAGGCTTTGAGCGAAGAGCTCAAAGATGAACTGGTTATCATCATGAGGGCCTACTTGGAAAAACCAAGAACTACCGTCGGATGGAAAGGTTTGATCAATGACCCAGATTTGGACAACAGTTTCAGCATCAATAAGGGTCTTAAGATTAGTAGAAAGTTGTTTGTTGACCTGACCAACAGCGGTATGCCTATTGCCAGTGAAATGTTGGACACAATTTCCCCTCAATACTTGGCTGATTTGCTATCTTTCGGTGCCATCGGTGCCAGAACAACTGAGTCACAGCTTCACAGAGAGCTGGCATCAGGACTTAGCTTCCCCATCGGATTCAAGAATGGAACTGATGGTACCTTGGATGTTGCTCTTGATGCTGTTCAAGCTGCTTCTCATGCTCATCACTTCATGGGTGTCACGAAGCACGGTATTGCTGCCATCACGACCACAAAGGGTAATGAATACTGTTTCGTCATTCTTAGAGGAGGTAAGAAGGGTACGAACTACGATCCTGAATCTGTGGCCGAGGCAAAGGCAAAGTTACCTGCTGACGGTGTTCTGATGATTGACTGTTCCCACGGAAACTCCAACAAGGATTACAGAAACCAACCAAAGGTCTCCAAGGTGGTTGCCGATCAGGTCGCTGCTGGTGAAGACCGTATTATTGGAGTAATGATCGAATCCAACATCAATGAAGGTAAGCAATCCATTCCAGCTGAAGGAAGAAAGGCTCTTAAGTATGGTGTTTCTGTCACTGACGGCTGTGTCTCGTGGGAAACTACCGACGCTATGTTGAGAGAACTTGCTGAAGCTGTTCAAAAAAGAAGAGCATTAAAGAACGCTGCTTAG

>*KpARO3* from *Komagataella phaffii* GS115 (*Pichia pastoris*)

ATGTTCATTCAAAACGATCATGTCGGTGACAGATCCCGCTTAGAAGACTGGAGAATACGTGGATACGACCCTCTGACGCCCCCCGACCTGTTGCAACATGAGTATCCACTTACCCCAAAAGCAGAGGAGAACATTCTCAAGGGAAGAAACGAAGCTGTTGATATCCTGAAAGGTAAGGATGACAGGCTATTAGTCATTGTCGGACCTTGTTCCATCCATGATCCTAAGGCAGCTTTAGACTACTGCGAGAGATTAGCCAAGTTTAACGAGACTATCAAAGGTGAGCTTCATATCATAATGAGAGCTTATTTGGAGAAACCAAGAACCACTGTTGGTTGGAAAGGTCTGATAAATGACCCAGACATCGATGGTTCGTTTCAAATCAATAAAGGTCTTCGTATCTCGAGGGAGCTTTTCGTTAAACTTACTGAACAGATTCCTATTGCTGGTGAGATGCTGGACACTATCTCCCCTCAATTCCTGTCTGATCTCTTCTCCTTAGGAGCGATTGGCGCCAGAACCACAGAGTCTCAACTGCACAGAGAACTTGCTTCCGGTTTGTCCTTCCCCGTGGGATTCAAGAATGGTACAGACGGTGGGCTCACAGTTGCTATAGATGCCATGAGAGCTGCTTCGCATCCTCACCATTTCCTGTCTGTCACAAAACCAGGAGTTGTGGCTATTGTCGGTACCGAAGGAAATGAAGACACCTTTGTTATCCTAAGAGGTGGTAAGAACGGTACAAACTACGACGAGGCCTCTGTTGACGCTGCCCACGCACAGTTAGAGAAGACCGGTATCCTGGGCTCTGGTCCTGCCATCATGGTGGACTGCTCTCATGGAAATTCCAACAAAGACCACCGTAACCAACCTAAGGTAGCCCAAGTTGTTGCTGACCAAATCCGTAAAGGATCCCGAAAAATATGTGGCCTCATGATTGAGAGCAATATCGTTGAAGGTAGACAGGATGTTCCCAAAGAGGGAAAGTCACATCTACGTTATGGATGTTCCATAACTGATGCCTGTATCTCCTGGGAGGATACTGAGAAGGTTCTACGAGTCTTAGCAGATTCGGTCATTGAACGTCGTAATCTCAAGAATTAG

>*aroF* from *Escherichia coli*

ATGCAAAAAGACGCGCTGAATAACGTACATATTACCGACGAACAGGTTTTAATGACTCCGGAACAACTGAAGGCCGCTTTTCCATTGAGCCTGCAACAAGAAGCCCAGATTGCTGACTCGCGTAAAAGCATTTCAGATATTATCGCCGGGCGCGATCCTCGTCTGCTGGTAGTATGTGGTCCTTGTTCCATTCATGATCCGGAAACTGCTCTGGAATATGCTCGTCGATTTAAAGCCCTTGCCGCAGAGGTCAGCGATAGCCTCTATCTGGTAATGCGCGTCTATTTTGAAAAACCCCGTACCACTGTCGGCTGGAAAGGGTTAATTAACGATCCCCATATGGATGGCTCTTTTGATGTAGAAGCCGGGCTGCAGATCGCGCGTAAATTGCTGCTTGAGCTGGTGAATATGGGACTGCCACTGGCGACGGAAGCGTTAGATCCGAATAGCCCGCAATACCTGGGCGATCTGTTTAGCTGGTCAGCAATTGGTGCTCGTACAACGGAATCGCAAACTCACCGTGAAATGGCCTCCGGGCTTTCCATGCCGGTTGGTTTTAAAAACGGCACCGACGGCAGTCTGGCAACAGCAATTAACGCTATGCGCGCCGCCGCCCAGCCGCACCGTTTTGTTGGCATTAACCAGGCAGGGCAGGTTGCGTTGCTACAAACTCAGGGGAATCCGGACGGCCATGTGATCCTGCGCGGTGGTAAAGCGCCGAACTATAGCCCTGCGGATGTTGCGCAATGTGAAAAAGAGATGGAACAGGCGGGACTGCGCCCGTCTCTGATGGTAGATTGCAGCCACGGTAATTCCAATAAAGATTATCGCCGTCAGCCTGCGGTGGCAGAATCCGTGGTTGCTCAAATCAAAGATGGCAATCGCTCAATTATTGGTCTGATGATCGAAAGTAATATCCACGAGGGCAATCAGTCTTCCGAGCAACCGCGCAGTGAAATGAAATACGGTGTATCCGTAACCGATGCCTGCATTAGCTGGGAAATGACCGATGCCTTGCTGCGTGAAATTCATCAGGATCTGAACGGGCAGCTGACGGCTCGCGTGGCTTAA
